# Maternal methylmercury exposure changes the proteomic profile of the offspring’s salivary glands: Prospects on translational toxicology

**DOI:** 10.1371/journal.pone.0258969

**Published:** 2021-11-08

**Authors:** Priscila Cunha Nascimento, Walessa Alana Bragança Aragão, Leonardo Oliveira Bittencourt, Aline Dionizio, Marilia A. R. Buzalaf, Maria Elena Crespo-Lopez, Rafael Rodrigues Lima

**Affiliations:** 1 Laboratory of Functional and Structural Biology, Institute of Biological Sciences, Federal University of Para, Belém, PA, Brazil; 2 Department of Biological Sciences, Bauru School of Dentistry, University of São Paulo, Bauru, São Paulo, Brazil; 3 Laboratory of Molecular Pharmacology, Institute of Biological Sciences, Federal University of Para, Belém, PA, Brazil; Chinese Academy of Sciences, CHINA

## Abstract

**Background:**

Methylmercury (MeHg) remains a public health issue since developing organisms are particularly vulnerable to this environmental contaminant. This study investigated the effect of maternal MeHg exposure on the modulation of proteomic profile of parotid (PA), submandibular (SM), and sublingual (SL) glands of offspring rats.

**Materials and methods:**

Pregnant Wistar rats were daily exposed to 40 μg/kg MeHg during both gestational and lactation periods. The proteomic profiles of the major salivary glands of the offspring rats were analyzed through mass spectrometry.

**Results:**

The offspring rats exposed to MeHg showed significant alterations in the proteomic profiles of the PA, SM, and SL glands. Altered proteins were associated with cytoskeleton components, tissue morphogenesis, and response to stimulus and stress.

**Conclusion:**

This original study showed that maternal MeHg exposure significantly modulates the expression of proteins and induces alterations in the proteomic profiles of developing salivary glands.

## Introduction

Developing organisms are particularly vulnerable to environmental toxins [1–4]. Methylmercury (MeHg) is a highly bioaccumulative toxic compound that remains a public health issue due to its natural and anthropogenic environmental distributions[4,5], such as soil erosion and biomass burning [6,7]. Primary (fossil fuel combustion, minerals, and waste) and secondary anthropogenic activities (Hg-based products and artisanal gold mining) are the main contributors to the global biogeochemical cycling of Hg and cause high levels of reemissions [1,8].

Several studies have shown that MeHg induces multiple systemic damages [9], mainly neurological impairments [4,7,10]. Our research group recently evidenced structural changes in the salivary glands of offspring rats after gestational and lactational MeHg exposure [11]. It is widely known that changes in the homeostasis of salivary glands can damage other oral tissues due to deficits in salivary production, composition, and molecular mechanisms involved in oral physiology [12–14].

It has been shown that the total Hg levels in the salivary glands of intoxicated offspring rats were associated with toxicopathologic findings evidenced by glandular morphometric changes and damages in both epithelial and myoepithelial architecture [11], which may decrease both the quality and quantity of saliva [15,16]. This fluid has been widely used in clinical and toxicological analyses of human metabolism [17,18] due to its easy and non-invasive collection and better cost efficiency for large-scale trials than the blood serum. However, the use of saliva as a diagnostic matrix for the analysis of MeHg environmental exposure is still unclear [19].

It seems relevant to explore the association between potential tissue or functional modifications and mechanisms of molecular modulation [20]; thus, this present study aimed to investigate the effect of maternal MeHg exposure on the modulation of proteins biomarkers in the parotid (PA), submandibular (SM), and sublingual (SL) glands of offspring rats.

## Materials and methods

This study was previously approved by the Ethics committee on animal experimentation by of Federal University of Pará (UFPA), under protocol number 8613011217/CEUA-UFPA and is available to check at http://ceua.ufpa.br/#, by “protocol status” tool. All the experiments followed a guide for the use of laboratory animals [21] and the Animal Research: Reporting of In Vivo Experiments (ARRIVE) guidelines [22] (S1 Table).

The datasets presented in this study can be found in online repositories. The names of the repository/repositories and accession number(s) can be found below: Peptide Atlas, accession no: PASS01692.

### Animals and experimental design

Pregnant Wistar rats (*Rattus norvegicus*), with 90-days-old and weighed 150 to 200g, were kept at UFPA vivarium under a 14:10h light and dark cycle (lights on 7:00 AM) and a climate-controlled room (25±2°C). The animals were kept in individual cages with food and water *ad libitum* in association with the experimental protocol.

**Fig 1:**
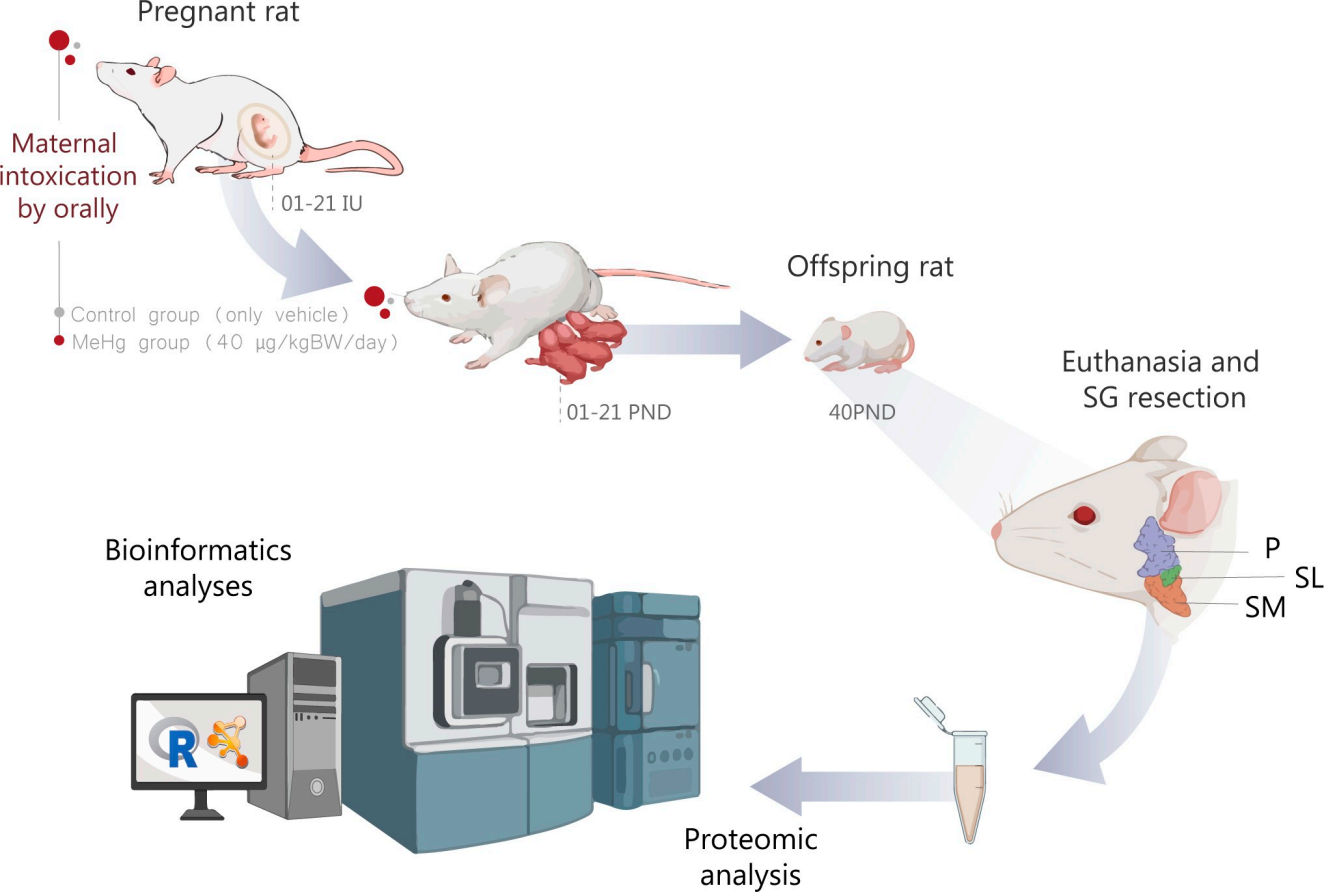
Flow chart of the study design. The animals were exposed to MeHg (40 μg/kg B.W./day) or only vehicle by maternal intoxication, from the 01 intrauterine day (IU) until 21 postnatal days (PND). After tissue maturation, at 40PND, the offspring rats were euthanized for salivary glands resection. Then, the Parotid, Submandibular (SM) Sublingual (SL) glands were processed for proteomic analyses by Mass Spectrometry analysis followed by bioinformatics analyses.

A sample size calculation for the number of progenitor animals was performed, assuming a normal distribution of the variables tested. A power of 80% and a bilateral alpha level of 5% were considered with mean and standard deviation data of a previous study and detailed in Nascimento et al. [11]. The flow chart of the study design is summarized in Fig 1.

### Protocol of MeHg intoxication

The pregnant female rats were equally and randomly distributed into two groups according to the experimental protocol: 1) Control group (n = 5): received vehicle only; 2) MeHg group (n = 5): received MeHg (40 μg/kg B.W./day) [23]. The animals were weekly weighed for dose adjustment (for review of weight data, see Nascimento et al. [11]).

The dissolution of methylmercury chloride (CH3HgCl; Sigma-Aldrich, Milwaukee, ‎Wisconsin, USA) and the administration by cookie treats (Teddy Grahams, Nabisco, CAN) were performed as previously described [11]. Briefly, the contaminated cookies were offered once a day, only to progenitor animals and were immediately eaten, as confirmed by a visual inspection. The administration occurred during the gestational (20–21 days) and lactation periods (20–21 days).

After 24 hours of the last MeHg administration (at the end of the lactation period), the progenitor female rats were euthanized by cervical dislocation under anesthesia, with a mixture of ketamine chloride and xylazine chloride (90 mg/kg and 9 mg/kg, respectively, i. p.). Then the surgery for the collection of larger salivary glands was performed. After the assessment, the Hg levels evidence the successful intoxication protocol, as observed and described by Nascimento et al. [11].

Parallelly, the offspring rats were divided by genders and maintained in collective cages (4 animals each) with food and water *ad libitum*, and kept in a climate-controlled room with a light/dark cycle appropriate until 40-days-old, once the salivary glands are entirely developed and thus possible to perform the analyses proposed [24].

At the 40-days-old, these pups were anaesthetized (with a mixture of ketamine and xylazine, as described above) for resection of the parotid, submandibular lingual glands for the subsequent proteomic analysis. The samples were immediately frozen in liquid nitrogen and stored at −80°C until evaluations. The researchers were blinded regarding the analyses of the groups.

### Proteomic profile

#### Preparation of salivary gland samples

All the steps of the proteomics approach following the protocols previously described [25,26]. Briefly, the analyses were performed by sample homogenization, protein extraction, reduction, alkylation, digestion, desalination, and purification. First, samples of larger salivary glands (parotid, submandibular and lingual glands) of the animals (n = 6 per group) were pooled 2 in 2, and the analyses were performed in biological triplicate. The sample size of 6 animals per group (3 pools from 2 animals each) has been widely used in proteomic studies involving quantitative analysis of different animal tissues [25,27–29]. By having three pools from each group, we have biological triplicates. In addition, each pool is run in the mass spectrometer three times (technical triplicates). Thus, we had nine mass spectrometer runs for each group, which is enough for proper statistical analysis using the Protein Lynx Global Server (PLGS) software embedded in the equipment.

For the homogenization, a buffer solution was added in the samples to extract soluble proteins with lysis buffer (7 M urea, 2 M thiourea, and 40 mM DTT diluted in AMBIC; BioRad, USA) under constant stirring at 4° C. Then, the samples were centrifuged (at 20,817 g / 4° C for 30 min), and the supernatant was collected for total protein quantification by Bradford’s method [30]. After that, AMBIC (50 mM) was added in 100 μg of protein up to 50 μL. In the next stage, each sample received 10 μL of AMBIC, and 25 μL of 0,2% RapiGEST ™ (Waters Co., Manchester, UK) and they were incubated (at 37°C for 30 min). At the end of this process, 5 mM DTT was added and incubated again (37° C for 40 min). After this, 10 mM IAA (BioRad, USA) was added and incubated for 30 min at room temperature and dark conditions.

For the digestion step, 10 μL of trypsin (Thermo Fischer, USA) was added to the samples (at 37° C for 14 h) followed by the addition of 10 μL of 5% trifluoroacetic acid (Sigma-Aldrich, USA) and incubated (at 37° C for 90 min). In the next step, supernatants were collected and purified using C18 spin columns (Pierce, USA). At the end of the procedure, the samples were resuspended in 12 mL of ADH (1 pmol mL1) associated with 108 mL of 3% acetonitrile (Sigma-Aldrich, USA) and 0.1% formic acid (Thermo Fischer, USA) to be submitted to Mass Spectrometry analysis.

#### Mass spectrometry analysis

The reading and identification of the peptides were conducted by a nanoAcquity UPLCXevo QTof MS system (Waters, Manchester, UK), using the Protein Lynx Global Server (PLGS), as previously detailed [31]. The proteins verification was determined by downloading the Uniprot database. Then, the bioinformatics analyses were performed using Cytoscape (3.6.1 version, Java®) with the ClueGO plugin for the determination of biological process groups, based on Gene Ontology (GO) annotations [32]. Additionally, the ClusterMarker plugin was for the protein-interaction network.

#### Over-representation analysis (ORA)

Initially, a table was built including ID code, name of the proteins and their respective Log2Ratio values. For proteins with absolute changes, -1 values were assigned for proteins detected only in control and 1 for proteins detected only in the MeHg group. For the ORA analysis, was used the R studio program [33] with the EGSEA plugin [34]. In this process, the UNIPROT database was accessed to identify proteins and the biological processes they participate in, made available by Bader Lab. After this verification, we used the Cytoscape software [35] with Enrichment Lab plugin pipeline for grouping the sets of proteins previously consulted. After that, the main biological processes were selected for graphical analysis. In this analysis, we used the Enrichment Map Cytoscape Plugin [35], where the list of protein sets with statistically significant changes was used as input. Then, networks and clusters were built to visualize the interconnections between the protein sets. The relationship between the different protein sets was constructed based on the similarity coefficient between them (Jaccard similarity coefficient = number of proteins in common between protein sets A and B/total number of proteins in A + B). In order to identify the clusters and their respective functions, the AutoAnnotate Cytoscape App [36] was used, which names each cluster. After that, the clusters were classified into larger categories of biological processes. Then, a PPI analysis (https://www.networkanalyst.ca/) [37] was carried to results in the representative image, according to the number of interactions of the proteins with the other proteins found altered. A minimum of 10 interactions was applied. The R studio program generated the image with the GOplot plugin.

#### Statistical analyses

All identified proteins were tabulated using electronic spreadsheets (Microsoft Excel®, Windows 10 version) and representation of performing the GO analyses. For comparative analysis, the PLGS software with the Monte-Carlo algorithm was applied and to obtain the difference of protein expression between the groups, p < 0.05 for down-regulated proteins and p > 0.95 for up-regulated proteins were considered.

## Results

### Salivary glands proteins profiling of offspring after pre- and postnatal exposure to methylmercury

The MeHg transfer from the mothers that consumed 40 μg/kg B.W./day MeHg (an exposure of environmental meaning) modulated the proteomic profile of salivary glands of offspring. This exposure model revealed a total of 201, 603, and 228 altered proteins in Parotid, Submandibular, and Sublingual glands, respectively. In the parotid gland, 37 proteins were uniquely regulated in the control group, and 41 were uniquely in the MeHg group (Table 1). The submandibular gland proteome revealed 177 uniquely expressed in the control group and 63 uniquely in the exposed group (Table 2). Also, when analyzing the Sublingual proteome, were observed 164 proteins expressed only in the MeHg group, and there were no exclusive expressed proteins in the control group, as observed the Table 3. The proteins roster complete is described in Supplementary Tables (S1–S3 Tables).

**Table 1:** Unique proteins in parotid gland of offspring rats after pre- and postnatal exposure to methylmercury vs control group.

Accession ID^a^	Description	*PLGS* Score	Control	MeHg
**P38983**	40S ribosomal protein SA	189.76	+	-
**Q63041**	Alpha-1-macroglobulin	34.36	+	-
**P24090**	Alpha-2-HS-glycoprotein	62.56	-	+
**Q02874**	Core histone macro-H2A.1	70.81	+	-
**P70623**	Fatty acid-binding protein_ adipocyte	544.58	-	+
**P04906**	Glutathione S-transferase P	81.61	+	-
**G3V7G8**	Glycine—tRNA ligase	30.37	+	-
**Q6IMY8**	Heterogeneous nuclear ribonucleoprotein U	149.07	+	-
**O35274**	Neurabin-2	35.6	-	+
**P13084**	Nucleophosmin	201.7	+	-
**P24368**	Peptidyl-prolyl cis-trans isomerase B	201.43	+	-
**Q6AYD3**	Proliferation-associated protein 2G4	66.87	+	-
**P35284**	Ras-related protein Rab-12	132.37	-	+
**P61107**	Ras-related protein Rab-14	132.37	-	+
**P51156**	Ras-related protein Rab-26	132.37	-	+
**Q53B90**	Ras-related protein Rab-43	132.37	-	+
**P07340**	Sodium/potassium-transporting ATPase subunit beta-1	179.01	+	-
**P16086**	Spectrin alpha chain_ non-erythrocytic 1	32.29	+	-
**D3ZSP7**	Tetratricopeptide repeat domain 3	17.24	-	+
**P63029**	Translationally-controlled tumor protein	133.74	+	-
	*+58 proteins exclusive of control or MeHg group*			

^a^ Accession ID according to the Uniport.org database.

Signs of + or indicate the presence or absence of the protein in one of the groups.

**Table 2:** Unique proteins in Submandibular gland of offspring rats after pre- and postnatal exposure to methylmercury (MeHg) vs. control group.

Accession ID^a^	Description	*PLGS* Score	Control	MeHg
**P29314**	40S ribosomal protein S9	357.01	+	-
**P62832**	60S ribosomal protein L23	326.57	+	-
**B5DF11**	AN1-type zinc finger protein 5	121.59	+	-
**P07150**	Annexin A1	45.02	-	+
**Q9WU82**	Catenin beta-1	60.64	+	-
**P36375**	Glandular kallikrein-10	288.36	+	-
**P36373**	Glandular kallikrein-7_ submandibular/renal	316.88	+	-
**P30713**	Glutathione S-transferase theta-2	431.7	-	+
**O55148**	Growth arrest-specific protein 7	73.71	+	-
**P25030**	Keratin_ type I cytoskeletal 20	87.88	+	-
**D3Z9H7**	Nuclear factor of activated T-cells_ cytoplasmic 4	95.45	-	+
**Q9JID1**	Programmed cell death protein 4	116.22	-	+
**Q9WVC0**	Septin-7	97.44	+	-
**Q920J4**	Thioredoxin-like protein 1	162.08	-	+
**Q63355**	Unconventional myosin-Ic	110.53	+	-
**Q8VDA5**	Z-DNA-binding protein 1	72.25	-	+
	*+224 proteins exclusive of control or MeHg group*			

^a^ Accession ID according to the Uniport.org database.

Signs of + or indicate the presence or absence of the protein in one of the groups.

**Table 3:** Unique proteins in sublingual gland of offspring rats after pre- and postnatal exposure to methylmercury (MeHg) vs control group.

Accession ID^a^	Description	*PLGS* Score	Control	MeHg
P04764	Alpha-enolase	671.14	-	+
P04906	Glutathione S-transferase P	118.93	-	+
Q63942	GTP-binding protein Rab-3D	102.33	-	+
P0DMW0	Heat shock 70 kDa protein 1A	12.25	-	+
P0DMW1	Heat shock 70 kDa protein 1B	12.25	-	+
Q5XHZ0	Heat shock protein 75 kDa_ mitochondrial	96.56	-	+
P82995	Heat shock protein HSP 90-alpha	113.77	-	+
P34058	Heat shock protein HSP 90-beta	109.19	-	+
Q63617	Hypoxia up-regulated protein 1	31.22	-	+
P30904	Macrophage migration inhibitory factor	1074.24	-	+
Q63716	Peroxiredoxin-1	184.22	-	+
P48721	Stress-70 protein_ mitochondrial	42.7	-	+
	*+153 proteins exclusive of the MeHg group*			

^a^ Accession ID according to the Uniport.org database.

Signs of + or indicate the presence or absence of the protein in one of the groups.

**Fig 2:**
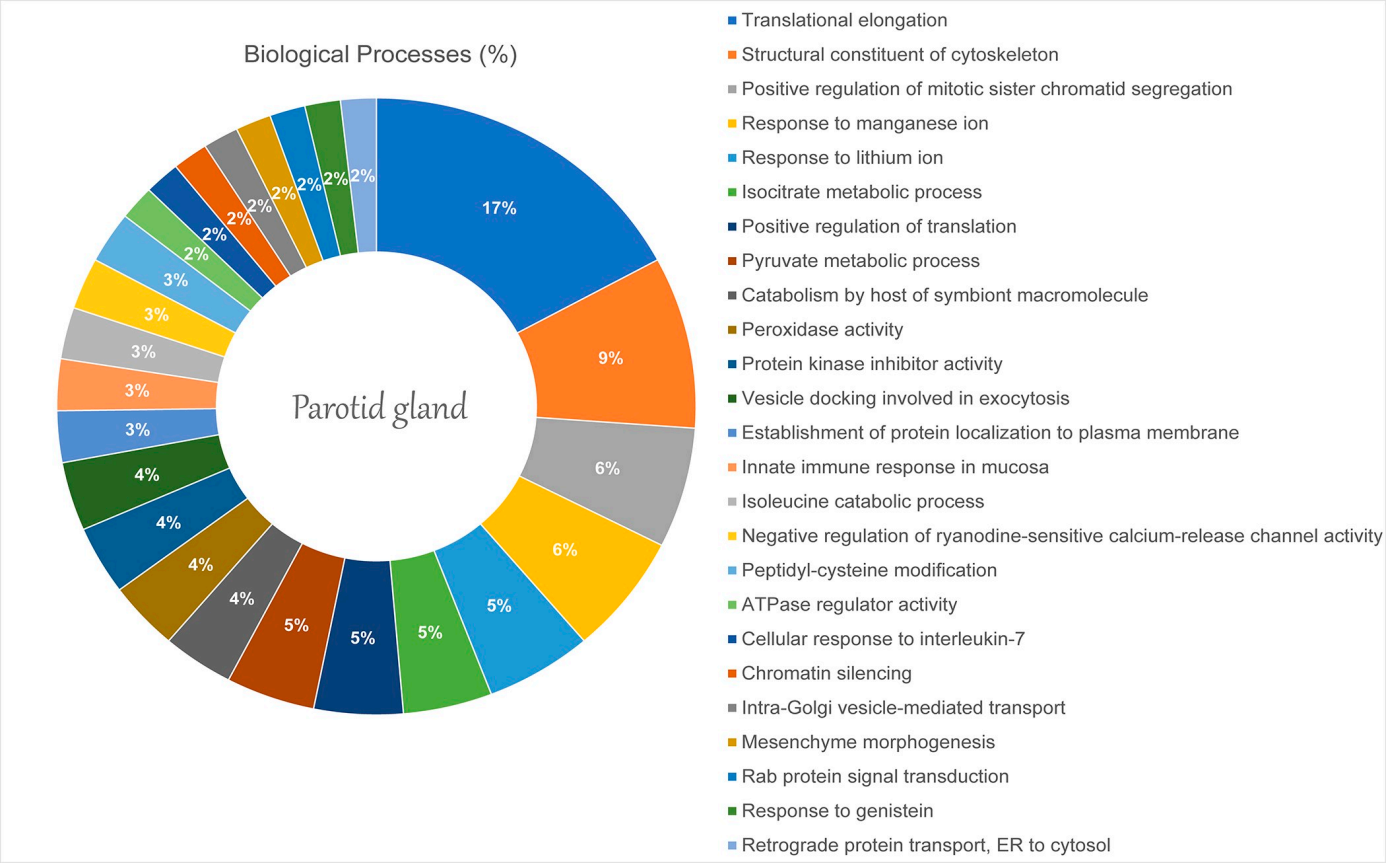
Functional distribution of proteins identified with differential expression proteins on the parotid gland of offspring rats after pre- and postnatal exposure to methylmercury vs. control group. The categories of proteins based on Gene Ontology annotation of biological process and the proteins access numbers were provided by UNIPROT. Terms significant (Kappa Score = 0.4) and distribution according to the percentage of the number of genes and was evaluated by ClueGO® plugin of Cytoscape® software 3.7.1.

**Fig 3:**
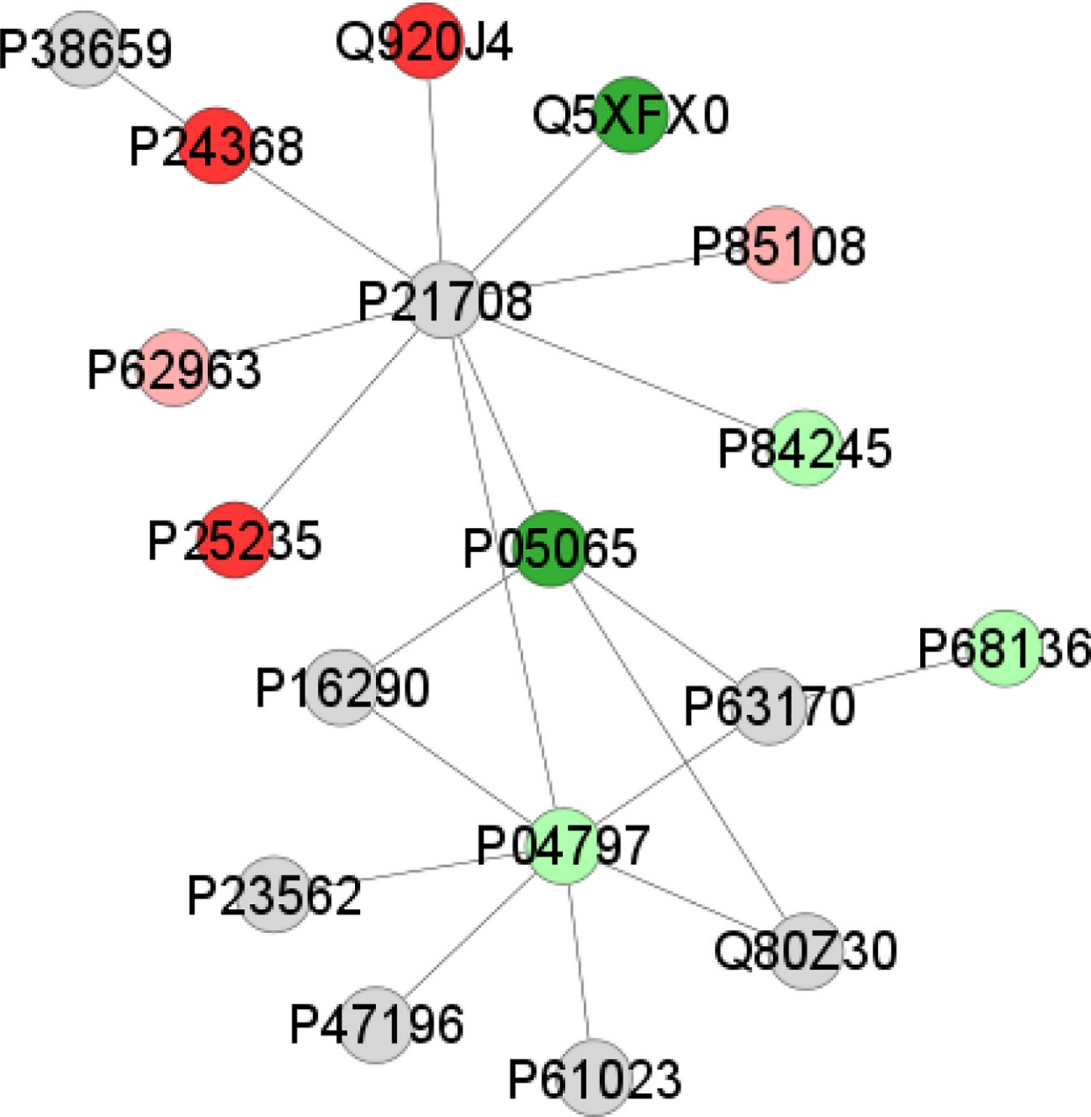
Subnetwork created in the ClusterMark app to identify protein-protein interactions (PPI) in parotid glands of the offspring exposed to MeHg. The proteins are identified according to the accession ID of the UNIPROT database. The node colors represent proteins with different statuses of regulation (light green: upregulated; pink: downregulated), proteins exclusive to the exposed group (green) and exclusive to control group (red). The gray nodes indicate proteins that were not identified in the samples, but that interacted through the analysis of the databases. Identification of the proteins found in the samples according to the accession ID: Q920J4 (Thiore-doxin-like protein 1), P24368 (Peptidyl-prolyl cis-trans isomerase B), P62963 (Profilin-1), P25235 (Doli-chyl-diphosphooligosaccharide—protein glycosyltransferase subunit 2), P05065 (Fructose-bisphosphate aldolase A), P04797 (Glyceraldehyde-3-phosphate dehydrogenase), P68136 (Actin, alpha skeletal muscle), P84245 (Histone H3.3), P85108 (Tubulin beta-2A chain), Q5XFX0 (Transalivary glandselin-2).

The pre- and postnatal exposure to environmental methylmercury also changed the salivary glands of offspring rats. According to Gene Ontology, 25 categories of biological processes were affected in the Parotid Gland: the translational elongation (17.12%) and the structural constituent of cytoskeleton (9.1%) were the most affected groups (Fig 2). The proteome networks analysis revealed the interaction of proteins related mainly to the cytoskeleton, such as Glyceraldehyde-3-phosphate dehydrogenase (P04797), Actin, alpha skeletal muscle (P68136), and Profilin-1 (P62963), Tubulin beta-2A chain (P85108) (Fig 3).

**Fig 4:**
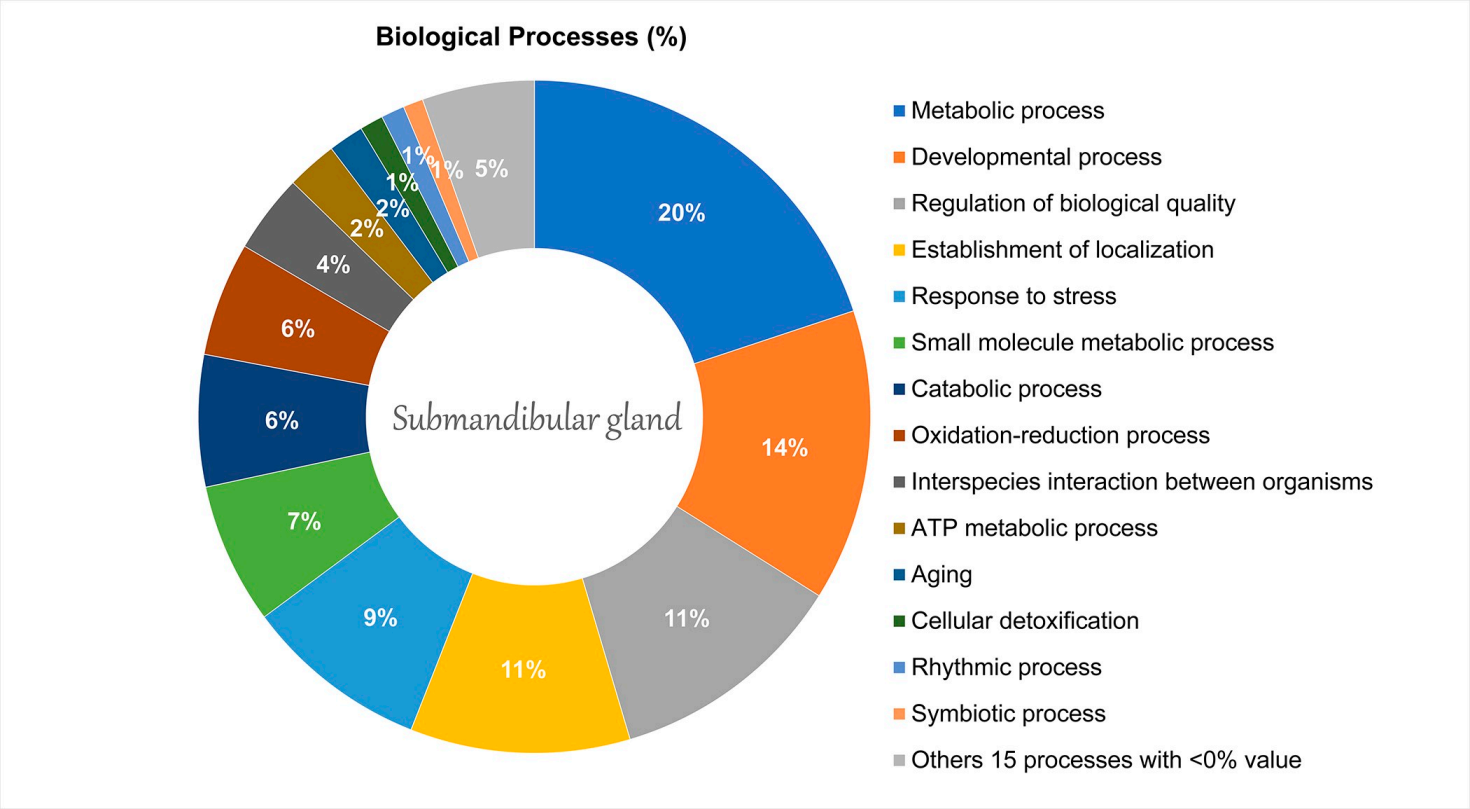
Functional distribution of proteins identified with differential expression proteins on Submandibular gland of offspring rats after pre- and postnatal exposure to methylmercury vs. control group. The categories of proteins based on Gene Ontology annotation of biological process and the proteins access number were provided by UNIPROT. Terms significant (Kappa Score = 0.4) and distribution according to the percentage of the number of genes and was evaluated by ClueGo® plugin of Cytoscape® software 3.7.1.

**Fig 5:**
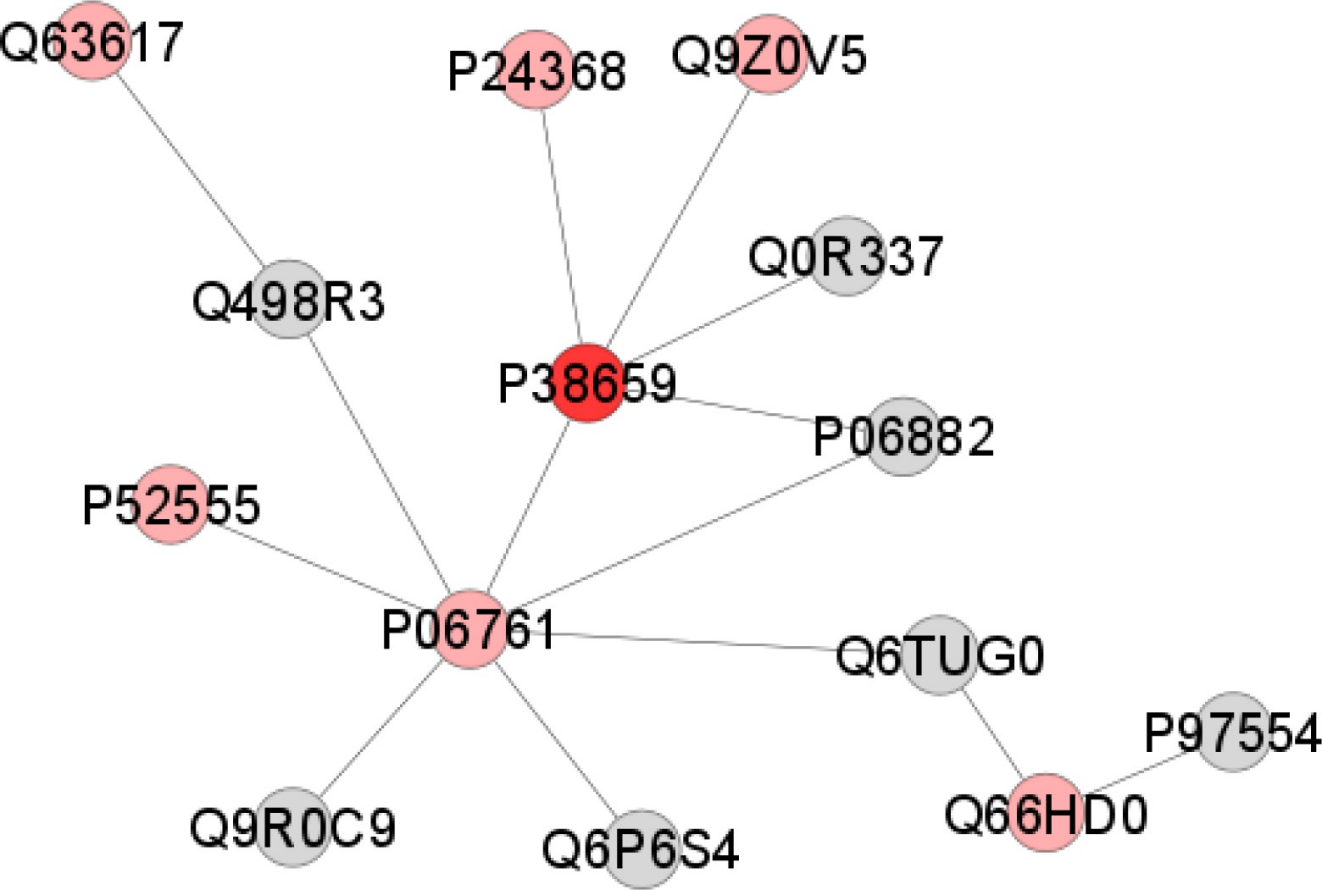
Subnetwork created in the ClusterMark app to identify protein-protein interactions (PPI) in submandibular glands of the off-spring exposed to MeHg. The proteins are identified according to the accession ID of the UNIPROT database. The colors of the nodes represent proteins with differences in expression (pink: downregulated) and proteins exclusive to the control group (red). The gray nodes indicate proteins that were not identified in the samples but interacted through the database analysis. Identification of the proteins found in the samples according to the accession ID: Q63617 (Hypoxia up-regulated protein 1), P52555 (Endoplasmic reticulum resident protein 29), P06761 (Endoplasmic reticulum chaperone BiP), P38659 (Protein disulfide-isomerase A4), Q66HD0 (Endoplasmin), Q9Z0V5 (Peroxiredoxin-4), P24368 (Peptidyl-prolyl cis-trans isomerase B).

In addition, the proteins related to the metabolic process (19.9%) and developmental process (14.01%) showed significant changes in the submandibular gland (Fig 4) The proteomics network revealed the interaction between proteins that are related to protein metabolism and changes in the cellular redox system, such as: Protein disulfide-isomerase A4 (P38659), Endoplasmic reticulum resident protein 29 (P52555), Endoplasmin (Q66HD0), Endoplasmic reticulum chaperone BiP (P06761), Peptidyl-prolyl cis-trans isomerase B (P24368), Hypoxia up-regulated protein 1 (Q63617), and Peroxiredoxin-4 (Q9Z0V5) (Fig 5).

**Fig 6:**
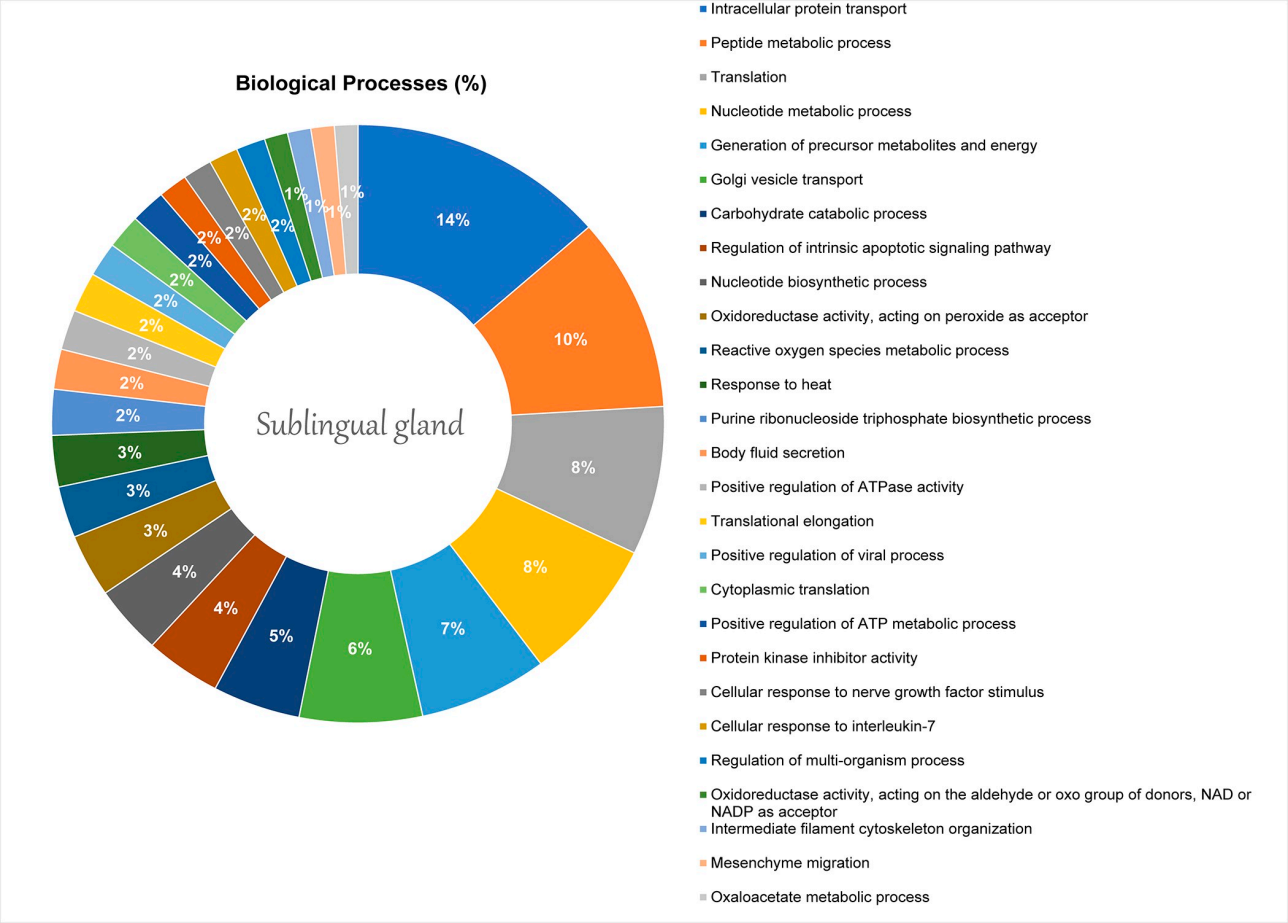
Functional distribution of proteins identified with differential expression proteins on Sublingual gland of offspring rats after pre- and postnatal exposure to methylmercury vs. control group. The categories of proteins based on Gene Ontology annotation of biological process and the proteins access number were provided by UNIPROT. Terms significant (Kappa Score = 0.4) and distribution according to the percentage of number of genes and was evaluated by ClueGo® plugin of Cytoscape® software 3.7.1.

**Fig 7:**
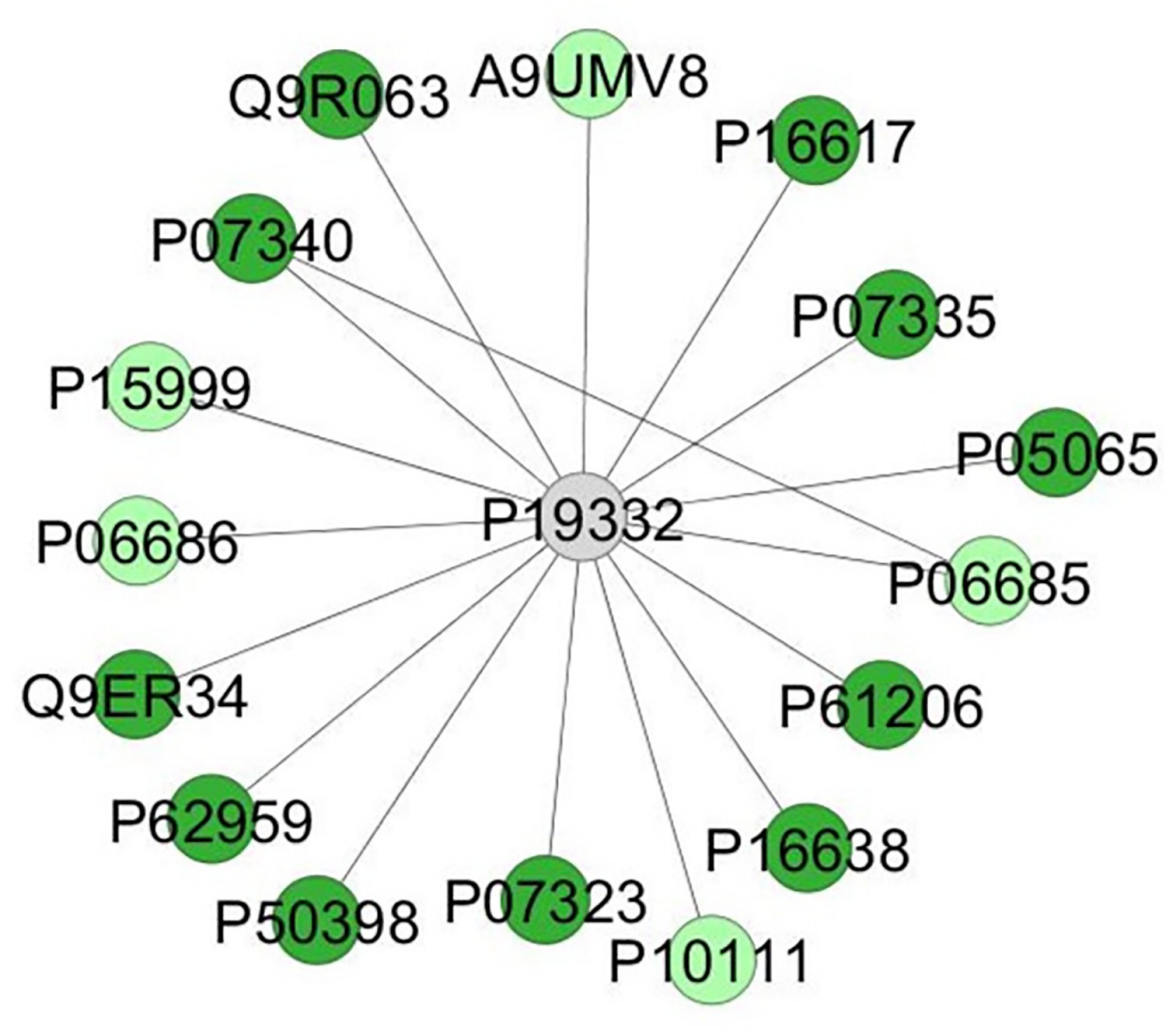
Subnetwork created in the ClusterMark app to identify protein-protein interactions (PPI) in sublingual glands of the offspring exposed to MeHg. The proteins are identified according to the accession ID of the UNIPROT database. The colors of the nodes represent proteins with differences in expression (light green: upregulated) and proteins exclusive to the exposed group (green). The gray nodes indicate proteins that were not identified in the samples but that interacted through the analysis of the databases. Identification of the proteins found in the samples according to the accession ID: Q9R063 (Peroxiredoxin-5, mitochondrial), P07340 (Sodium/potassium-transporting ATPase subunit beta-1), P15999 (ATP synthase subunit alpha, mitochondrial), P06686 (Sodi-um/potassium-transporting ATPase subunit alpha-2), Q9ER34 (Aconitate hydratase, mitochondrial), P62959 (Histidine triad nucleotide-binding protein 1), P50398 (Rab GDP dissociation inhibitor alpha), P07323 (Gamma-enolase), P10111 (Peptidyl-prolyl cis-trans isomerase A), P16638 (ATP-citrate synthase), P61206 (ADP-ribosylation factor 3), P06685 (Sodium/potassium-transporting ATPase subunit alpha-1), P05065 (Fructose-bisphosphate aldolase A), P07335 (Creatine kinase B-type), P16617 (Phosphoglycerate kinase 1), A9UMV8 (Histone H2A.J).

While in the sublingual, the main altered biological processes were about intracellular protein transport (13.58%) and peptide metabolic process (10.49%), as detailed in Fig 6. The proteomic network showed the interaction between proteins mainly related to the mitochondrial activity (Fig 7), such as Peroxiredoxin-5, mitochondrial (Q9R063), ATP synthase subunit alpha, mitochondrial (P15999), Sodium/potassium-transporting ATPase subunit alpha-2 (P06686), Aconitate hydratase, mitochondrial (Q9ER34), and Peptidyl-prolyl cis-trans isomerase A (P10111).

### Early exposure to methylmercury modulates significantly the salivary glands proteomic profile of offspring rats

**Fig 8:**
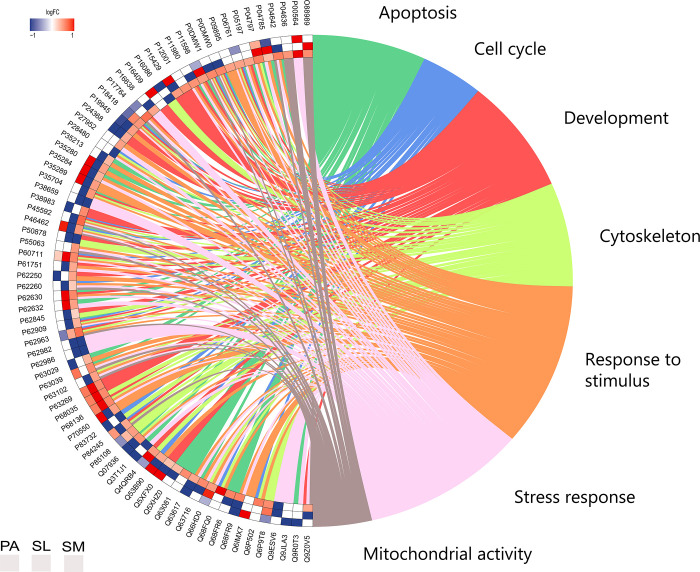
Circos plot of protein-protein interaction (PPI). The analysis of the Parotid (PA), Sublingual (SL), and Submandibular (SM) glands of offspring rats after pre- and postnatal exposure to methylmercury (MeHg) vs. control group in categories apoptosis (green), cell cycle (blue), development (red) cytoskeleton (lime green), response to stimulus (orange), stress response (pink) and mitochondrial activity (grey). Blue-scale proteins are down-regulated and red up-regulated in the MeHg group when compared to the control group.

In quantitative analyses between the groups, changed expression proteins were down-regulated or up-regulated, consisting of 51 proteins with different status of regulation on parotid, 314 on submandibular, and 54 on sublingual glands (S4–S6 Tables). The over-representation analysis (ORA) resulted in 76 proteins, allowing us to observe the interactions that each protein presented in each exposed group between proteins up or down-regulated, and the proteins with exclusive regulation in each group, as shown in Fig 8.

## Discussion

This original study showed molecular changes in the salivary glands of offspring rats caused by maternal MeHg exposure. Developing salivary glands seem a target for MeHg toxicity since significant changes in the modulation of proteins associated with cytoskeleton components, tissue morphogenesis, and stimulus/stress response was observed. This study aimed to identify alterations at the protein level and correlate them with previously reported morphological changes [11].

Humans are mainly exposed to MeHg through the consumption of contaminated fish and seafood [4,38,39]. Even if mothers are not affected, MeHg can be transferred to fetuses during pregnancy and lactation and induce long-term adverse effects on developing organs. Alterations in neonates and children induced by maternal MeHg exposure have been observed in chronically exposed populations such as Amazonian rivers [10]. Therefore, this exposure model aimed to mimic the daily intake of MeHg contaminated food by pregnant women. The determination of a safe level of MeHg exposure is complex, especially for vulnerable groups such as fetuses and infants. Although the World Health Organization (WHO) indicates 1.6 μg/kg/BW MeHg as the tolerable weekly intake (PTWI) [40], chronically exposed populations are usually found with higher MeHg levels [14–16]. Recent biomonitoring has revealed hair Hg levels as high as 75 ppm [41–43], which may represent a MeHg weekly intake of 52.5 μg/kg as suggested by Crespo-Lopez [1]. Thus, the MeHg dose used in this study (40 μg/kg/BW) resembles the exposure detected in chronically exposed humans and is widely used in validated research with rats [11,23,44–46]

Organic Hg is mainly absorbed by the gastrointestinal tract, enters into the blood circulation, and is rapidly distributed to other tissues [6,39]. Due to its high lipophilicity, MeHg can immediately cross biological barriers; thus, MeHg consumed by pregnant women easily crosses the placenta [6,47]. The central nervous system is traditionally reported as the main target for MeHg and its neurotoxin mechanisms have been investigated by several studies [1]; however, MeHg can also damage other systems, such as immunological, renal, and cardiovascular [1,10]. Recent findings of our research group indicated that MeHg induced alterations in oral cell lines [48], alveolar bone [49], and salivary glands [11,44–46]. Since developing salivary glands significantly accumulate Hg [11], saliva can be potentially used as a matrix for biomonitoring environmental human exposure to MeHg [13]. This study results determined the proteins involved in the modulation of the salivary glands of offspring rats after pre-and postnatal MeHg exposure and thus contribute to validate the saliva as an additional diagnostic tool.

Increasing evidence has shown that significant environmental MeHg levels can change biological functions in humans and animals; however, adverse molecular implications are not yet fully understood [20]. Therefore, this data described the association between Hg accumulation in offspring rats and the modulation of the salivary gland proteomic profiles. A total of 1032 proteins were found altered in the three salivary gland pairs (S1–S6 Tables). When compared to a study with adult animals [45], the offspring rats evaluated in this study interestingly presented a greater number of altered proteins, which suggests that early exposure to MeHg is more harmful. Moreover, the abovementioned intoxication model may have strengthened the findings of this study.

Our research group has previously, observed that the PA glands were more susceptible to Hg accumulation than SM and SL glands when evaluated under the same MeHg exposure protocol [11]; however, PA glands were the second most affected in terms of proteins number, which, suggests that the cells present in these glands play a role on defense mechanisms against MeHg. This study indicated that Hg levels mainly induced alterations in the biological process associated with translational elongation (Fig 1), such as the 40S ribosomal protein (*P38983*) not expressed in the MeHg group that may lead to tissue morphogenesis [50]. The structural constituent of the cytoskeleton was also changed and can be explained by previous immunohistochemistry findings [11]. In addition, the proteomics network analysis showed some protein-protein interactions that participate in cytoskeleton cellular processes, such as the up-regulation of glyceraldehyde-3-phosphate dehydrogenase (*P04797*) and actin alpha skeletal muscle (*P68136*), which indicates the modulation of PA cytoskeleton as a response to MeHg stimuli. By contrast, the downregulation of proteins associated with actin filaments and microtubules formation [51,52] such as profilin-1 (P62963) and tubulin beta-2A chain (P85108) indicates an impairment in the maintenance of cytoskeleton components that jeopardizes the cell morphology.

Regarding the proteomic changes of SM glands, a total of 603 altered proteins among 27 biological processes were observed. Both metabolic (20%) and developmental (14%) processes were the most affected by MeHg, while kallikreins (glandular kallikrein-10, *P36375*; glandular kallikrein-7_submandibular/renal, *P36373*) and annexin (*P07150*) were the most altered protein groups. The kallikreins directly maintain oral health by acting as vasodilatation mediators in damaged oral mucosa, which favor both defense and cicatricial processes [53]; in addition, they indirectly protect and repair dental enamel against dental caries due to proteolytic activation of proteins, such as the proline-rich protein [54]. The association of kallikreins with survival and proliferation cells has also been suggested since these proteins bind to glandular receptors that may activate critical survival pathways such as PI3K-Akt [55]. Moreover, kallikreins are predominantly found in the striated ducts and ductal cells of mammals’ SM glands [56,57]. Therefore, the lack of kallikreins expression in the MeHg group of this study may compromise the regeneration capacity of the SM glands and also explain previous MeHg-induced toxicopathologic findings in the ductal area [11].

The proteomic analysis showed the expression of annexins only in the SM glands of offspring rats exposed to MeHg (Table 2). These calcium-binding proteins can be involved in intracellular transport [58] and some family members such as annexin A1 (ANXA1, P07150) are associated with the inflammatory process [59]. Moreover, ANXA1 has an important functional attribute in the phagocytic clearance of apoptotic cells [59]. It is well known that Hg exposure leads to inflammation [60]; however, this study specifically describes the molecular pathways of damage associated with MeHg exposure in the salivary glands of offspring rats. The oxidative stress was also a pathway of damage associated with MeHg exposure. The proteomic network analysis revealed protein-protein interactions related to protein metabolism. The disulfide isomerase A4 protein(*P38659*), which controls disulfide bonds between two thiol groups, was exclusively observed in the group control. The down-regulation of endoplasmic reticulum resident protein 29 (*P52555*), endoplasmin (*Q66HD0*), and endoplasmic reticulum chaperone BiP (*P06761*) associated with secretory proteins processing in the endoplasmic reticulum (ER) can compromise protein folding and cell survival [61]. Conversely, the downregulation of peptidyl-prolyl cis-trans isomerase B (*P24368*), hypoxia up-regulated protein 1 (*Q63617*), and peroxiredoxin-4 (*Q9Z0V5*) proteins indicate changes in redox homeostasis control. It must be highlighted that peroxiredoxin-4 is responsible for cellular protection against oxidative stress by reducing hydrogen peroxide in water and alcohol [5]; thus, its downregulation favors cellular oxidative reactions.

Similarly, the results of SL glands analysis also emphasized the role of oxidative stress mechanisms in MeHg intoxication of developing salivary glands. Interestingly, a lack of protein expression was observed in the control group (Table 3), while the majority of proteins expressed in the MeHg group was related to stress response such as the heat shock proteins (HSP) 70 kDa 1A [P0DMW0], HSP 70 kDa 1B [P0DMW1], HSP 75 kDa mitochondrial [Q5XHZ0], HSP 90-alpha [P82995], and HSP 90-beta [P34058]). The HSP is produced as a response to stress conditions such as exposure to toxins [62]. The findings of this study with offspring rats are similar to those found in a study with adult animals exposed to MeHg and corroborate with the oxidative imbalance evidenced by Bittencourt et al. [45].

Furthermore, proteins associate with stimulus-response were only expressed in the SL glands exposed to MeHg. The alpha-enolase (P04764) presents different functions and can be associated with HSP and cytoskeletal structures [64]. This protein was recently indicated as an antigenic target in primary Sjogren’s syndrome [65], which is an autoimmune disease mainly associated with xerostomia; thus, this study findings regarding salivary glands exposed to MeHg may be directly related to oral dryness [66]. The proteomic network analysis of the MeHg group revealed protein-protein interactions such as peroxiredoxin-5 mitochondrial (Q9R063) and aconitate hydratase mitochondrial (Q9ER34) in the mitochondrial activity, which suggests a response to oxidative reactions induced by reactive oxygen species (ROS). Meanwhile, the ATP synthase subunit alpha mitochondrial (P15999), sodium/potassium-transporting ATPase subunit alpha-2 (P06686), and peptidyl-prolyl cis-trans isomerase A (P10111) proteins were up-regulated, which indicates increased mitochondrial activity as a cellular response to MeHg toxicity.

Overall, the main protein categories affected by MeHg in the salivary glands are related to apoptosis, cell cycle, development, cytoskeleton, stimulus-response, stress, and mitochondrial activity (Fig 4). Considering the previously reported MeHg-induced structural changes in salivary glands, a down-regulation of the proteins associated with glandular architecture was expected; however, the variations observed in the over-representation analysis need a more detailed investigation. A subexpression of the actin gamma-enteric smooth muscle (*P63269*), actin alpha cardiac muscle 1 (*P68035*), and actin alpha skeletal muscle (*P68136*) proteins was observed in all salivary glands. The three main actin isoforms (alpha, beta, and gamma) have been identified in vertebrate animals. The alpha actins are found in muscle tissues and are significant constituents of the contractile apparatus, while both beta and gamma actins coexist as cytoskeleton components in most cell types and are mediators of internal cell motility [63].

The modulation of proteins associated with the ubiquitin-proteasome system, which is vital for proteostasis, was observed in all salivary glands. The abovementioned HSP are associated with maturation, re-folding, and degradation of proteins [64,65]. The HSP 70-binding 1 (Q6IMX7) was only expressed in PA glands exposed to MeHg, while the HSP 10 kDa mitochondrial (P26772) was only expressed in the SM glands of the control group and HSP with different molecular weights such as 10 kDa (P26772; exclusive in control), 70 kDa, and 90 kDa (P0DMW0 and P82995, respectively) were only observed in the SL glands exposed to MeHg. Moreover, the E3 ubiquitin-protein ligase TRIM23 (P36407) was only observed in the SM glands exposed to MeHg, the polyubiquitin-B (P0CG51) was only expressed in the SL glands exposed to MeHg, and the ubiquitin-fold modifier 1 (Q5BJP3), which act as a quality control [66,67], was only observed in the control PA glands. Therefore, the potential proteostasis impairment can cause protein aggregation or dysfunctionality, and compromise glandular activity.

Overall, this study suggests that salivary glands are targets for MeHg-induced molecular changes during early life, especially at an environmental exposure dose. This experimental–environmental model demonstrated the main proteomic changes in PA, SM, and SL glands associated with significant modulation of proteins involved in cytoskeleton components, tissue morphogenesis, and response to stimulus and stress. Therefore, further studies are needed to investigate whether such changes can also alter saliva biomarkers in vulnerable populations such as neonates and infants.

## Supporting information

S1 Table Unique proteins in parotid gland of offspring rats of the MeHg group vs. control group. (DOCX)

S2 Table Unique proteins in submandibular gland of offspring rats of the MeHg group vs. control group. (DOCX)

S3 Table Unique proteins in sublingual gland of offspring rats of the MeHg group vs. control group. (DOCX)

S4 Table Identified proteins with significantly different expression altered in Parotid Gland of offspring rats of the MeHg group vs. control group. (DOCX)

S5 Table Identified proteins with significantly different expression altered in submandibular gland of offspring rats MeHg group vs. control group. (DOCX)

S6 Table Identified proteins with significantly different expression altered in sublingual gland of offspring rats of the MeHg group vs. control group. (DOCX)

S7 Table The ARRIVE guidelines checklist. (DOCX)
