# Supplementary material for: Maternal methylmercury exposure changes the proteomic profile of the offspring’s salivary glands: Prospects on translational toxicology
Source: PLoS One. 2021 Nov 8;16(11):e0258969. doi: 10.1371/journal.pone.0258969 (PMC8575261; doi:10.1371/journal.pone.0258969)
Supplement: S1 Table — (DOCX) [file pone.0258969.s001.docx]

**Table S1.** Unique proteins in Parotid Gland of offspring rats of the MeHg group vs. control group

| Accession ID^a^ | Description | *PLGS*  Score | Group |
| --- | --- | --- | --- |
| P63324 | 40S ribosomal protein S12 | 681.63 | Control |
| P62250 | 40S ribosomal protein S16 | 1166.79 | Control |
| P38983 | 40S ribosomal protein SA | 189.76 | Control |
| P02401 | 60S acidic ribosomal protein P2 | 644.68 | Control |
| P41123 | 60S ribosomal protein L13 | 209.36 | Control |
| P83732 | 60S ribosomal protein L24 | 624.1 | Control |
| Q63041 | Alpha-1-macroglobulin | 34.36 | Control |
| P07150 | Annexin A1 | 239.24 | Control |
| Q07936 | Annexin A2 | 138.39 | Control |
| B0BNL6 | Arrestin domain-containing protein 1 | 58.31 | Control |
| P49088 | Asparagine synthetase [glutamine-hydrolyzing] | 46.16 | Control |
| O35854 | Branched-chain-amino-acid aminotransferase_ mitochondrial | 59.71 | Control |
| P24268 | Cathepsin D | 295.53 | Control |
| P23514 | Coatomer subunit beta | 36.98 | Control |
| Q02874 | Core histone macro-H2A.1 | 70.81 | Control |
| Q9R0T3 | DnaJ homolog subfamily C member 3 | 151.92 | Control |
| P25235 | Dolichyl-diphosphooligosaccharide--protein glycosyltransferase subunit 2 | 81.35 | Control |
| Q68FR9 | Elongation factor 1-delta | 122.38 | Control |
| Q3T1J1 | Eukaryotic translation initiation factor 5A-1 | 587.08 | Control |
| P08010 | Glutathione S-transferase Mu 2 | 1351.46 | Control |
| P04906 | Glutathione S-transferase P | 81.61 | Control |
| G3V7G8 | Glycine--tRNA ligase | 30.37 | Control |
| Q7TP47 | Heterogeneous nuclear ribonucleoprotein Q | 59.65 | Control |
| Q6IMY8 | Heterogeneous nuclear ribonucleoprotein U | 149.07 | Control |
| Q63617 | Hypoxia up-regulated protein 1 | 92 | Control |
| P13084 | Nucleophosmin | 201.7 | Control |
| P24368 | Peptidyl-prolyl cis-trans isomerase B | 201.43 | Control |
| Q6AYD3 | Proliferation-associated protein 2G4 | 66.87 | Control |
| D4ACX8 | Protocadherin-16 | 23.24 | Control |
| P07340 | Sodium/potassium-transporting ATPase subunit beta-1 | 179.01 | Control |
| P16086 | Spectrin alpha chain_ non-erythrocytic 1 | 32.29 | Control |
| Q920J4 | Thioredoxin-like protein 1 | 86.62 | Control |
| P63029 | Translationally-controlled tumor protein | 133.74 | Control |
| Q5I0E7 | Transmembrane emp24 domain-containing protein 9 | 559.53 | Control |
| Q64428 | Trifunctional enzyme subunit alpha_ mitochondrial | 82.56 | Control |
| Q5BJP3 | Ubiquitin-fold modifier 1 | 547.24 | Control |
| Q9JLA3 | UDP-glucose:glycoprotein glucosyltransferase 1 | 62.17 | Control |
| P62243 | 40S ribosomal protein S8 | 82.87 | MeHg |
| P47198 | 60S ribosomal protein L22 | 135.98 | MeHg |
| Q9ER34 | Aconitate hydratase_ mitochondrial | 142.66 | MeHg |
| P24090 | Alpha-2-HS-glycoprotein | 62.56 | MeHg |
| P15429 | Beta-enolase | 95.88 | MeHg |
| A0JPN3 | BPI fold-containing family B member 1 | 88.3 | MeHg |
| P0DP29 | Calmodulin-1 | 753.64 | MeHg |
| P0DP30 | Calmodulin-2 | 753.64 | MeHg |
| P0DP31 | Calmodulin-3 | 753.64 | MeHg |
| P00564 | Creatine kinase M-type | 104.75 | MeHg |
| P23965 | Enoyl-CoA delta isomerase 1_ mitochondrial | 200.4 | MeHg |
| P70623 | Fatty acid-binding protein_ adipocyte | 544.58 | MeHg |
| P05065 | Fructose-bisphosphate aldolase A | 64.19 | MeHg |
| P09117 | Fructose-bisphosphate aldolase C | 168.04 | MeHg |
| Q66H61 | Glutamine--tRNA ligase | 54.76 | MeHg |
| Q63942 | GTP-binding protein Rab-3D | 132.37 | MeHg |
| Q6IMX7 | Hsp70-binding protein 1 | 49.73 | MeHg |
| P02600 | Myosin light chain 1/3_ skeletal muscle isoform | 517.19 | MeHg |
| P16409 | Myosin light chain 3 | 16.8 | MeHg |
| P12847 | Myosin-3 | 42.24 | MeHg |
| O35274 | Neurabin-2 | 35.6 | MeHg |
| P04182 | Ornithine aminotransferase_ mitochondrial | 863.09 | MeHg |
| P35281 | Ras-related protein Rab-10 | 366.01 | MeHg |
| P35284 | Ras-related protein Rab-12 | 132.37 | MeHg |
| P35286 | Ras-related protein Rab-13 | 233.63 | MeHg |
| P61107 | Ras-related protein Rab-14 | 132.37 | MeHg |
| P35289 | Ras-related protein Rab-15 | 347.81 | MeHg |
| P10536 | Ras-related protein Rab-1B | 366.01 | MeHg |
| P51156 | Ras-related protein Rab-26 | 132.37 | MeHg |
| Q5U316 | Ras-related protein Rab-35 | 355.96 | MeHg |
| P63012 | Ras-related protein Rab-3A | 132.37 | MeHg |
| Q63941 | Ras-related protein Rab-3B | 132.37 | MeHg |
| P62824 | Ras-related protein Rab-3C | 132.37 | MeHg |
| Q53B90 | Ras-related protein Rab-43 | 132.37 | MeHg |
| P05714 | Ras-related protein Rab-4A | 132.37 | MeHg |
| P51146 | Ras-related protein Rab-4B | 132.37 | MeHg |
| Q9WVB1 | Ras-related protein Rab-6A | 314.65 | MeHg |
| P35280 | Ras-related protein Rab-8A | 366.01 | MeHg |
| P70550 | Ras-related protein Rab-8B | 366.01 | MeHg |
| D3ZSP7 | Tetratricopeptide repeat domain 3 | 17.24 | MeHg |
| Q5XFX0 | Transgelin-2 | 260.47 | MeHg |

^a^ Accession ID according to the Uniport.org database.
