# Supplementary material for: Maternal methylmercury exposure changes the proteomic profile of the offspring’s salivary glands: Prospects on translational toxicology
Source: PLoS One. 2021 Nov 8;16(11):e0258969. doi: 10.1371/journal.pone.0258969 (PMC8575261; doi:10.1371/journal.pone.0258969)
Supplement: S2 Table — (DOCX) [file pone.0258969.s002.docx]

**Table S2.** Unique proteins in Submandibular Gland of offspring rats of the MeHg group vs. control group

| Accession ID^a^ | Description | *PLGS*  Score | Group |
| --- | --- | --- | --- |
| P26772 | 10 kDa heat shock protein_ mitochondrial | 910.74 | Control |
| B0BN93 | 26S proteasome non-ATPase regulatory subunit 13 | 197.04 | Control |
| Q62904 | 3-keto-steroid reductase | 153.47 | Control |
| P62282 | 40S ribosomal protein S11 | 157.55 | Control |
| P62271 | 40S ribosomal protein S18 | 61.4 | Control |
| P60868 | 40S ribosomal protein S20 | 110.78 | Control |
| P05765 | 40S ribosomal protein S21 | 680.21 | Control |
| P62859 | 40S ribosomal protein S28 | 1285.5 | Control |
| P62864 | 40S ribosomal protein S30 | 1498.26 | Control |
| P62703 | 40S ribosomal protein S4_ X isoform | 124.96 | Control |
| P29314 | 40S ribosomal protein S9 | 357.01 | Control |
| Q9JLJ3 | 4-trimethylaminobutyraldehyde dehydrogenase | 133 | Control |
| P23358 | 60S ribosomal protein L12 | 559.68 | Control |
| P41123 | 60S ribosomal protein L13 | 276.02 | Control |
| P62832 | 60S ribosomal protein L23 | 326.57 | Control |
| P83732 | 60S ribosomal protein L24 | 452.39 | Control |
| P50878 | 60S ribosomal protein L4 | 381.58 | Control |
| P21533 | 60S ribosomal protein L6 | 143.37 | Control |
| P85971 | 6-phosphogluconolactonase | 477.97 | Control |
| P29410 | Adenylate kinase 2_ mitochondrial | 166.09 | Control |
| P17475 | Alpha-1-antiproteinase | 267.92 | Control |
| Q66H12 | Alpha-N-acetylgalactosaminidase | 130.01 | Control |
| Q6AYS7 | Aminoacylase-1A | 502.77 | Control |
| Q6PTT0 | Aminoacylase-1B | 387.44 | Control |
| B5DF11 | AN1-type zinc finger protein 5 | 121.59 | Control |
| Q62893 | Anti-Muellerian hormone type-2 receptor | 118.77 | Control |
| P19511 | ATP synthase F(0) complex subunit B1_ mitochondrial | 358.09 | Control |
| P35434 | ATP synthase subunit delta_ mitochondrial | 159 | Control |
| Q5U216 | ATP-dependent RNA helicase DDX39A | 49.77 | Control |
| P59241 | Aurora kinase A | 122.31 | Control |
| O35567 | Bifunctional purine biosynthesis protein PURH | 64.56 | Control |
| Q5BK10 | Calpain-13 | 46.55 | Control |
| P14141 | Carbonic anhydrase 3 | 440.68 | Control |
| Q9WU82 | Catenin beta-1 | 60.64 | Control |
| Q8CFN2 | Cell division control protein 42 homolog | 107.29 | Control |
| Q62834 | Cell division cycle protein 123 homolog | 672.8 | Control |
| Q4V8A2 | Cell division cycle protein 27 homolog | 35.82 | Control |
| Q6MG61 | Chloride intracellular channel protein 1 | 104.15 | Control |
| O35142 | Coatomer subunit beta' | 69.14 | Control |
| Q66H80 | Coatomer subunit delta | 107.27 | Control |
| D4ABY2 | Coatomer subunit gamma-2 | 65.89 | Control |
| Q62930 | Complement component C9 | 94.19 | Control |
| Q6P4Z9 | COP9 signalosome complex subunit 8 | 186.71 | Control |
| P09605 | Creatine kinase S-type_ mitochondrial | 60.97 | Control |
| Q641Z4 | Cyclin-dependent kinase 9 | 89.11 | Control |
| P12075 | Cytochrome c oxidase subunit 5B_ mitochondrial | 449.83 | Control |
| P10818 | Cytochrome c oxidase subunit 6A1_ mitochondrial | 205.57 | Control |
| P35171 | Cytochrome c oxidase subunit 7A2_ mitochondrial | 643.64 | Control |
| P38650 | Cytoplasmic dynein 1 heavy chain 1 | 52.4 | Control |
| O08651 | D-3-phosphoglycerate dehydrogenase | 116.4 | Control |
| Q62651 | Delta(3_5)-Delta(2_4)-dienoyl-CoA isomerase_ mitochondrial | 278.77 | Control |
| Q01205 | Dihydrolipoyllysine-residue succinyltransferase component of 2-oxoglutarate dehydrogenase complex_ mitochondrial | 123.78 | Control |
| Q62696 | Disks large homolog 1 | 60.16 | Control |
| P31016 | Disks large homolog 4 | 99.24 | Control |
| P25235 | Dolichyl-diphosphooligosaccharide--protein glycosyltransferase subunit 2 | 116.13 | Control |
| Q6AYH5 | Dynactin subunit 2 | 81.76 | Control |
| D3ZDI6 | E3 ubiquitin-protein ligase MYLIP | 78.53 | Control |
| P68101 | Eukaryotic translation initiation factor 2 subunit 1 | 100.76 | Control |
| Q6P9U8 | Eukaryotic translation initiation factor 3 subunit H | 147.62 | Control |
| Q3T1J1 | Eukaryotic translation initiation factor 5A-1 | 601.41 | Control |
| O54921 | Exocyst complex component 2 | 126.32 | Control |
| Q5XI32 | F-actin-capping protein subunit beta | 568.3 | Control |
| P05369 | Farnesyl pyrophosphate synthase | 171.89 | Control |
| P12785 | Fatty acid synthase | 72 | Control |
| Q8VII0 | FERM domain-containing protein 6 (Fragment) | 206.1 | Control |
| P02680 | Fibrinogen gamma chain | 84.51 | Control |
| Q9Z1N1 | Fructose-1_6-bisphosphatase isozyme 2 | 72.8 | Control |
| Q9Z0U4 | Gamma-aminobutyric acid type B receptor subunit 1 | 43.73 | Control |
| P28234 | Gap junction alpha-5 protein | 142.71 | Control |
| P36375 | Glandular kallikrein-10 | 288.36 | Control |
| P36373 | Glandular kallikrein-7_ submandibular/renal | 316.88 | Control |
| P47819 | Glial fibrillary acidic protein | 79.63 | Control |
| P70627 | Glutamate carboxypeptidase 2 | 80.34 | Control |
| P10860 | Glutamate dehydrogenase 1_ mitochondrial | 88.87 | Control |
| Q4KMC4 | Glutamine--fructose-6-phosphate aminotransferase [isomerizing] 2 | 57.47 | Control |
| Q9Z1B2 | Glutathione S-transferase Mu 5 | 50.21 | Control |
| Q9ESV6 | Glyceraldehyde-3-phosphate dehydrogenase_ testis-specific | 90.34 | Control |
| P09811 | Glycogen phosphorylase_ liver form | 133.96 | Control |
| P09812 | Glycogen phosphorylase_ muscle form | 84.61 | Control |
| Q9R064 | Golgi reassembly-stacking protein 2 | 76.42 | Control |
| O55148 | Growth arrest-specific protein 7 | 73.71 | Control |
| Q8K586 | GTP-binding nuclear protein Ran_ testis-specific isoform | 224.26 | Control |
| Q5HZY2 | GTP-binding protein SAR1b | 852.15 | Control |
| P20059 | Hemopexin | 156.42 | Control |
| Q6URK4 | Heterogeneous nuclear ribonucleoprotein A3 | 221.3 | Control |
| Q9JJ54 | Heterogeneous nuclear ribonucleoprotein D0 | 263.04 | Control |
| A7VJC2 | Heterogeneous nuclear ribonucleoproteins A2/B1 | 367.9 | Control |
| B5DFK3 | Homeobox protein Hox-D9 | 162.73 | Control |
| Q9WVK7 | Hydroxyacyl-coenzyme A dehydrogenase_ mitochondrial | 606.17 | Control |
| P97519 | Hydroxymethylglutaryl-CoA lyase_ mitochondrial | 91.58 | Control |
| P20759 | Ig gamma-1 chain C region | 92.95 | Control |
| P20760 | Ig gamma-2A chain C region | 339.26 | Control |
| Q3B7D8 | Inactive ubiquitin thioesterase OTULINL | 125.38 | Control |
| P41562 | Isocitrate dehydrogenase [NADP] cytoplasmic | 81.51 | Control |
| Q5XIK7 | Katanin p60 ATPase-containing subunit A-like 1 | 121.99 | Control |
| P25030 | Keratin_ type I cytoskeletal 20 | 87.88 | Control |
| Q6IG03 | Keratin_ type II cytoskeletal 73 | 79.63 | Control |
| Q5SGE0 | Leucine-rich PPR motif-containing protein_ mitochondrial | 54.67 | Control |
| Q64573 | Liver carboxylesterase 4 | 60.6 | Control |
| Q63010 | Liver carboxylesterase B-1 | 79.11 | Control |
| P18163 | Long-chain-fatty-acid--CoA ligase 1 | 108.35 | Control |
| P51886 | Lumican | 125.46 | Control |
| Q5XIM7 | Lysine--tRNA ligase | 90.87 | Control |
| P02761 | Major urinary protein | 1570.68 | Control |
| P57113 | Maleylacetoacetate isomerase | 169.5 | Control |
| Q8VHF0 | MAP/microtubule affinity-regulating kinase 3 | 187.85 | Control |
| Q561Q8 | Mediator of RNA polymerase II transcription subunit 4 | 99.61 | Control |
| P08503 | Medium-chain specific acyl-CoA dehydrogenase_ mitochondrial | 139.47 | Control |
| F1LU71 | Methylglutaconyl-CoA hydratase_ mitochondrial | 78.53 | Control |
| P19332 | Microtubule-associated protein tau | 95.68 | Control |
| Q8K5B3 | Multiple coagulation factor deficiency protein 2 homolog | 578.29 | Control |
| Q64122 | Myosin regulatory light polypeptide 9 | 116.6 | Control |
| P69060 | N-acylneuraminate cytidylyltransferase | 112.53 | Control |
| P19234 | NADH dehydrogenase [ubiquinone] flavoprotein 2_ mitochondrial | 240.54 | Control |
| Q5EB81 | NADH-cytochrome b5 reductase 1 | 94.79 | Control |
| Q5BJX0 | N-terminal Xaa-Pro-Lys N-methyltransferase 1 | 73.22 | Control |
| Q63083 | Nucleobindin-1 | 69.47 | Control |
| Q9JI85 | Nucleobindin-2 | 239.62 | Control |
| P13383 | Nucleolin | 99.37 | Control |
| Q65Z14 | Oncostatin-M-specific receptor subunit beta | 57.35 | Control |
| P02625 | Parvalbumin alpha | 140.48 | Control |
| P14925 | Peptidylglycine alpha-amidating monooxygenase | 102.1 | Control |
| O35244 | Peroxiredoxin-6 | 188.07 | Control |
| P97535 | Phospholipase A1 member A | 76.49 | Control |
| D3ZW91 | POC1 centriolar protein homolog B | 92.65 | Control |
| P0CG51 | Polyubiquitin-B | 871.01 | Control |
| Q63429 | Polyubiquitin-C | 871.01 | Control |
| P10960 | Prosaposin | 90.23 | Control |
| P43114 | Prostaglandin E2 receptor EP4 subtype | 66.14 | Control |
| P40112 | Proteasome subunit beta type-3 | 263.53 | Control |
| P34067 | Proteasome subunit beta type-4 | 100.1 | Control |
| Q6MGD0 | Protein CutA | 222.87 | Control |
| P38659 | Protein disulfide-isomerase A4 | 135.31 | Control |
| Q6B345 | Protein S100-A11 | 613.41 | Control |
| P05964 | Protein S100-A6 | 970.03 | Control |
| Q63945 | Protein SET | 296.99 | Control |
| Q8K4S3 | Proton-associated sugar transporter A | 66.53 | Control |
| Q7TNY7 | Rab effector MyRIP | 88.38 | Control |
| P62494 | Ras-related protein Rab-11A | 156.87 | Control |
| O35509 | Ras-related protein Rab-11B | 156.87 | Control |
| P29315 | Ribonuclease inhibitor | 162.51 | Control |
| Q3T1I9 | RNA polymerase II-associated protein 1 | 50.82 | Control |
| P11507 | Sarcoplasmic/endoplasmic reticulum calcium ATPase 2 | 60.84 | Control |
| Q62991 | Sec1 family domain-containing protein 1 | 66.17 | Control |
| Q9Z143 | Semaphorin-4F | 73.64 | Control |
| D3ZTD8 | Semaphorin-5A | 73.33 | Control |
| Q9WVC0 | Septin-7 | 97.44 | Control |
| P09006 | Serine protease inhibitor A3N | 78.98 | Control |
| P57760 | Serine/threonine-protein kinase 16 | 89.13 | Control |
| O08678 | Serine/threonine-protein kinase MARK1 | 187.85 | Control |
| O08679 | Serine/threonine-protein kinase MARK2 | 198.02 | Control |
| P62138 | Serine/threonine-protein phosphatase PP1-alpha catalytic subunit | 150.09 | Control |
| P62142 | Serine/threonine-protein phosphatase PP1-beta catalytic subunit | 150.09 | Control |
| P63088 | Serine/threonine-protein phosphatase PP1-gamma catalytic subunit | 150.09 | Control |
| P29457 | Serpin H1 | 135.53 | Control |
| Q9WTR7 | Signal peptidase complex catalytic subunit SEC11C | 114.55 | Control |
| P16086 | Spectrin alpha chain_ non-erythrocytic 1 | 50.16 | Control |
| O35814 | Stress-induced-phosphoprotein 1 | 82.19 | Control |
| P07632 | Superoxide dismutase [Cu-Zn] | 99.54 | Control |
| P07895 | Superoxide dismutase [Mn]_ mitochondrial | 460.29 | Control |
| P28480 | T-complex protein 1 subunit alpha | 82.92 | Control |
| Q9Z0V6 | Thioredoxin-dependent peroxide reductase_ mitochondrial | 267.5 | Control |
| P24329 | Thiosulfate sulfurtransferase | 242.3 | Control |
| P61589 | Transforming protein RhoA | 309.08 | Control |
| Q63584 | Transmembrane emp24 domain-containing protein 10 | 417.48 | Control |
| Q68FR5 | Transmembrane protein 209 | 145.77 | Control |
| Q6AYT3 | tRNA-splicing ligase RtcB homolog | 188.61 | Control |
| P62982 | Ubiquitin-40S ribosomal protein S27a | 871.01 | Control |
| P62986 | Ubiquitin-60S ribosomal protein L40 | 871.01 | Control |
| Q5M7A4 | Ubiquitin-like modifier-activating enzyme 5 | 115.34 | Control |
| Q63355 | Unconventional myosin-Ic | 110.53 | Control |
| Q9QYG8 | Uridine-cytidine kinase 2 | 136.23 | Control |
| B2RZ78 | Vacuolar protein sorting-associated protein 29 | 172.75 | Control |
| P0C0A2 | Vacuolar protein-sorting-associated protein 36 | 126.04 | Control |
| Q3KR53 | Vasculin-like protein 1 | 61.95 | Control |
| Q5FVN8 | WD repeat_ SAM and U-box domain-containing protein 1 | 99.91 | Control |
| Q6AXZ5 | Zinc finger MYND domain-containing protein 10 | 99.9 | Control |
| P97608 | 5-oxoprolinase | 58.07 | MeHg |
| Q99NB7 | Acetyl-coenzyme A thioesterase | 62.98 | MeHg |
| P49911 | Acidic leucine-rich nuclear phosphoprotein 32 family member A | 146.74 | MeHg |
| Q6RY07 | Acidic mammalian chitinase | 445.42 | MeHg |
| Q7TN78 | Acyl-coenzyme A synthetase ACSM4_ mitochondrial | 56.36 | MeHg |
| P25409 | Alanine aminotransferase 1 | 84.26 | MeHg |
| P00763 | Anionic trypsin-2 | 88.29 | MeHg |
| O70511 | Ankyrin-3 | 38.53 | MeHg |
| P07150 | Annexin A1 | 45.02 | MeHg |
| O08701 | Arginase-2_ mitochondrial | 99.87 | MeHg |
| Q0ZHH6 | Atlastin-3 | 52.89 | MeHg |
| Q641Y8 | ATP-dependent RNA helicase DDX1 | 72.32 | MeHg |
| Q9ESS6 | Basal cell adhesion molecule | 78.48 | MeHg |
| Q6UPE0 | Choline dehydrogenase_ mitochondrial | 82.88 | MeHg |
| Q68FP9 | Conserved oligomeric Golgi complex subunit 6 | 73.74 | MeHg |
| P35353 | Corticotropin-releasing factor receptor 1 | 181.79 | MeHg |
| P00564 | Creatine kinase M-type | 328.94 | MeHg |
| Q5I0H5 | Cyclin-L2 | 97.83 | MeHg |
| Q99P39 | Cysteine desulfurase_ mitochondrial | 56.64 | MeHg |
| P12020 | Cysteine-rich secretory protein 1 | 760.96 | MeHg |
| Q64559 | Cytosolic acyl coenzyme A thioester hydrolase | 52.93 | MeHg |
| Q2VUH7 | Dixin | 68.01 | MeHg |
| P21575 | Dynamin-1 | 69.36 | MeHg |
| P36407 | E3 ubiquitin-protein ligase TRIM23 | 78.08 | MeHg |
| Q8R491 | EH domain-containing protein 3 | 66.59 | MeHg |
| P0C0K7 | Ephrin type-B receptor 6 | 34.62 | MeHg |
| B5DEH2 | Erlin-2 | 74.62 | MeHg |
| B2GUZ5 | F-actin-capping protein subunit alpha-1 | 45.78 | MeHg |
| Q924K2 | FAS-associated factor 1 | 53.06 | MeHg |
| P30713 | Glutathione S-transferase theta-2 | 431.7 | MeHg |
| P62959 | Histidine triad nucleotide-binding protein 1 | 403.33 | MeHg |
| D4AD37 | Inositol monophosphatase 3 | 50.49 | MeHg |
| P57790 | Kelch-like ECH-associated protein 1 | 91.51 | MeHg |
| Q6QLM7 | Kinesin heavy chain isoform 5A | 45.47 | MeHg |
| Q2PQA9 | Kinesin-1 heavy chain | 32.33 | MeHg |
| P09650 | Mast cell protease 1 | 290.91 | MeHg |
| Q4V8B3 | Mediator of RNA polymerase II transcription subunit 24 | 32.48 | MeHg |
| P41243 | Megakaryocyte-associated tyrosine-protein kinase | 64.36 | MeHg |
| Q6Y1S1 | Myeloid differentiation primary response protein MyD88 | 90.19 | MeHg |
| P12847 | Myosin-3 | 76.36 | MeHg |
| Q29RW1 | Myosin-4 | 320.51 | MeHg |
| P02563 | Myosin-6 | 62.16 | MeHg |
| P02564 | Myosin-7 | 66.8 | MeHg |
| Q5KTC7 | N-acylethanolamine-hydrolyzing acid amidase | 71.83 | MeHg |
| P12390 | Neuronal acetylcholine receptor subunit beta-2 | 49.44 | MeHg |
| D3Z9H7 | Nuclear factor of activated T-cells_ cytoplasmic 4 | 95.45 | MeHg |
| Q9QZR8 | PDZ domain-containing protein 2 | 48 | MeHg |
| Q2M2R8 | Peroxisomal targeting signal 1 receptor | 49.64 | MeHg |
| Q63737 | Phosducin-like protein | 59.53 | MeHg |
| Q9QWS8 | Potassium voltage-gated channel subfamily H member 8 | 64.98 | MeHg |
| Q9JID1 | Programmed cell death protein 4 | 116.22 | MeHg |
| Q6AYD3 | Proliferation-associated protein 2G4 | 69.55 | MeHg |
| P48004 | Proteasome subunit alpha type-7 | 84.92 | MeHg |
| Q6TLK4 | Rho GTPase-activating protein 27 | 50.15 | MeHg |
| Q8CJ99 | Sodium channel and clathrin linker 1 | 56.58 | MeHg |
| D3ZSP7 | Tetratricopeptide repeat domain 3 | 28.64 | MeHg |
| Q920J4 | Thioredoxin-like protein 1 | 162.08 | MeHg |
| Q925B3 | Transient receptor potential cation channel subfamily M member 7 | 40.89 | MeHg |
| P70566 | Tropomodulin-2 | 68.61 | MeHg |
| Q63610 | Tropomyosin alpha-3 chain | 55.48 | MeHg |
| Q7M767 | Ubiquitin-conjugating enzyme E2 variant 2 | 230.72 | MeHg |
| Q63615 | Vacuolar protein sorting-associated protein 33A | 123.9 | MeHg |
| Q8VDA5 | Z-DNA-binding protein 1 | 72.25 | MeHg |

^a^ Accession ID according to the Uniport.org database.
