# Supplementary material for: Maternal methylmercury exposure changes the proteomic profile of the offspring’s salivary glands: Prospects on translational toxicology
Source: PLoS One. 2021 Nov 8;16(11):e0258969. doi: 10.1371/journal.pone.0258969 (PMC8575261; doi:10.1371/journal.pone.0258969)
Supplement: S3 Table — (DOCX) [file pone.0258969.s003.docx]

**Table S3.** Unique proteins in Sublingual Gland of offspring rats of the MeHg group vs. control group

| Accession ID^a^ | Description | *PLGS* Score | Group |
| --- | --- | --- | --- |
| P35213 | 14-3-3 protein beta/alpha | 424.45 | MeHg |
| P62260 | 14-3-3 protein epsilon | 461.32 | MeHg |
| P68511 | 14-3-3 protein eta | 424.45 | MeHg |
| P61983 | 14-3-3 protein gamma | 432.01 | MeHg |
| P68255 | 14-3-3 protein theta | 173.05 | MeHg |
| P63102 | 14-3-3 protein zeta/delta | 929.15 | MeHg |
| Q64591 | 2_4-dienoyl-CoA reductase_ mitochondrial | 77.26 | MeHg |
| O70351 | 3-hydroxyacyl-CoA dehydrogenase type-2 | 136.49 | MeHg |
| P62282 | 40S ribosomal protein S11 | 554.71 | MeHg |
| P62845 | 40S ribosomal protein S15 | 482.73 | MeHg |
| P62246 | 40S ribosomal protein S15a | 1073.74 | MeHg |
| P04644 | 40S ribosomal protein S17 | 609.06 | MeHg |
| Q71TY3 | 40S ribosomal protein S27 | 875.47 | MeHg |
| P24051 | 40S ribosomal protein S27-like | 875.47 | MeHg |
| P62909 | 40S ribosomal protein S3 | 137.25 | MeHg |
| P62243 | 40S ribosomal protein S8 | 123.11 | MeHg |
| P97608 | 5-oxoprolinase | 54.43 | MeHg |
| P19945 | 60S acidic ribosomal protein P0 | 279.71 | MeHg |
| P02401 | 60S acidic ribosomal protein P2 | 604.69 | MeHg |
| P62914 | 60S ribosomal protein L11 | 387.96 | MeHg |
| P20280 | 60S ribosomal protein L21 | 578.25 | MeHg |
| P47198 | 60S ribosomal protein L22 | 487.44 | MeHg |
| P62890 | 60S ribosomal protein L30 | 1168.27 | MeHg |
| P50878 | 60S ribosomal protein L4 | 89.71 | MeHg |
| P17077 | 60S ribosomal protein L9 | 146.29 | MeHg |
| Q6RY07 | Acidic mammalian chitinase | 1203.3 | MeHg |
| Q9ER34 | Aconitate hydratase_ mitochondrial | 143.78 | MeHg |
| P11030 | Acyl-CoA-binding protein | 472.04 | MeHg |
| Q08163 | Adenylyl cyclase-associated protein 1 | 74.5 | MeHg |
| P84079 | ADP-ribosylation factor 1 | 262.03 | MeHg |
| P84082 | ADP-ribosylation factor 2 | 193.24 | MeHg |
| P61206 | ADP-ribosylation factor 3 | 262.03 | MeHg |
| P61751 | ADP-ribosylation factor 4 | 193.24 | MeHg |
| P84083 | ADP-ribosylation factor 5 | 193.24 | MeHg |
| P11884 | Aldehyde dehydrogenase_ mitochondrial | 121.65 | MeHg |
| P24090 | Alpha-2-HS-glycoprotein | 237.58 | MeHg |
| Q64057 | Alpha-aminoadipic semialdehyde dehydrogenase | 84.37 | MeHg |
| P04764 | Alpha-enolase | 671.14 | MeHg |
| P07150 | Annexin A1 | 178.78 | MeHg |
| Q07936 | Annexin A2 | 177.36 | MeHg |
| P14668 | Annexin A5 | 265.06 | MeHg |
| P48037 | Annexin A6 | 38.74 | MeHg |
| P04639 | Apolipoprotein A-I | 151 | MeHg |
| P49088 | Asparagine synthetase [glutamine-hydrolyzing] | 50.17 | MeHg |
| P13221 | Aspartate aminotransferase_ cytoplasmic | 55.47 | MeHg |
| P00507 | Aspartate aminotransferase_ mitochondrial | 111.36 | MeHg |
| Q06647 | ATP synthase subunit O_ mitochondrial | 162.74 | MeHg |
| P16638 | ATP-citrate synthase | 50.38 | MeHg |
| P15429 | Beta-enolase | 51.12 | MeHg |
| P11442 | Clathrin heavy chain 1 | 82.8 | MeHg |
| Q66H80 | Coatomer subunit delta | 54.78 | MeHg |
| Q4AEF8 | Coatomer subunit gamma-1 | 97.59 | MeHg |
| P45592 | Cofilin-1 | 177.08 | MeHg |
| P02454 | Collagen alpha-1(I) chain | 47.11 | MeHg |
| P07335 | Creatine kinase B-type | 116.43 | MeHg |
| Q5I0H5 | Cyclin-L2 | 65.87 | MeHg |
| P00406 | Cytochrome c oxidase subunit 2 | 247.89 | MeHg |
| P62898 | Cytochrome c_ somatic | 667.37 | MeHg |
| P80254 | D-dopachrome decarboxylase | 841.56 | MeHg |
| Q7M0E3 | Destrin | 156.47 | MeHg |
| P25235 | Dolichyl-diphosphooligosaccharide--protein glycosyltransferase subunit 2 | 205.66 | MeHg |
| P13803 | Electron transfer flavoprotein subunit alpha_ mitochondrial | 560.86 | MeHg |
| Q68FR9 | Elongation factor 1-delta | 107.47 | MeHg |
| Q68FR6 | Elongation factor 1-gamma | 103.26 | MeHg |
| Q5U2Q3 | Ester hydrolase C11orf54 homolog | 215.85 | MeHg |
| P05065 | Fructose-bisphosphate aldolase A | 83.69 | MeHg |
| P07323 | Gamma-enolase | 67.62 | MeHg |
| P09606 | Glutamine synthetase | 230.73 | MeHg |
| Q66H61 | Glutamine--tRNA ligase | 41.84 | MeHg |
| P08010 | Glutathione S-transferase Mu 2 | 658.77 | MeHg |
| P04906 | Glutathione S-transferase P | 118.93 | MeHg |
| P08009 | Glutathione S-transferase Yb-3 | 37.96 | MeHg |
| Q9ESV6 | Glyceraldehyde-3-phosphate dehydrogenase_ testis-specific | 52.17 | MeHg |
| G3V7G8 | Glycine--tRNA ligase | 59.48 | MeHg |
| Q63942 | GTP-binding protein Rab-3D | 102.33 | MeHg |
| P0DMW0 | Heat shock 70 kDa protein 1A | 12.25 | MeHg |
| P0DMW1 | Heat shock 70 kDa protein 1B | 12.25 | MeHg |
| Q5XHZ0 | Heat shock protein 75 kDa_ mitochondrial | 96.56 | MeHg |
| P82995 | Heat shock protein HSP 90-alpha | 113.77 | MeHg |
| P34058 | Heat shock protein HSP 90-beta | 109.19 | MeHg |
| Q794E4 | Heterogeneous nuclear ribonucleoprotein F | 51.91 | MeHg |
| Q8VHV7 | Heterogeneous nuclear ribonucleoprotein H | 71.9 | MeHg |
| Q6AY09 | Heterogeneous nuclear ribonucleoprotein H2 | 45.02 | MeHg |
| P61980 | Heterogeneous nuclear ribonucleoprotein K | 50.47 | MeHg |
| Q6IMY8 | Heterogeneous nuclear ribonucleoprotein U | 40.48 | MeHg |
| P62959 | Histidine triad nucleotide-binding protein 1 | 122.58 | MeHg |
| Q6LED0 | Histone H3.1 O | 929.43 | MeHg |
| P84245 | Histone H3.3 | 7632.21 | MeHg |
| Q63617 | Hypoxia up-regulated protein 1 | 31.22 | MeHg |
| P41562 | Isocitrate dehydrogenase [NADP] cytoplasmic | 42.66 | MeHg |
| Q6IFW5 | Keratin_ type I cytoskeletal 12 | 117.63 | MeHg |
| Q6IFV1 | Keratin_ type I cytoskeletal 14 | 117.63 | MeHg |
| Q6IFV3 | Keratin_ type I cytoskeletal 15 | 117.63 | MeHg |
| Q6IFU8 | Keratin_ type I cytoskeletal 17 | 122.9 | MeHg |
| Q6IFU7 | Keratin_ type I cytoskeletal 42 | 117.63 | MeHg |
| Q6P6Q2 | Keratin_ type II cytoskeletal 5 | 171.1 | MeHg |
| Q4FZU2 | Keratin_ type II cytoskeletal 6A | 171.1 | MeHg |
| Q6IG12 | Keratin_ type II cytoskeletal 7 | 564.19 | MeHg |
| Q6IG05 | Keratin_ type II cytoskeletal 75 | 195.86 | MeHg |
| Q9Z2H4 | Leucine-rich repeat-containing G-protein coupled receptor 4 | 36.99 | MeHg |
| P04642 | L-lactate dehydrogenase A chain | 148.53 | MeHg |
| P30904 | Macrophage migration inhibitory factor | 1074.24 | MeHg |
| Q03626 | Murinoglobulin-1 | 30.38 | MeHg |
| Q64119 | Myosin light polypeptide 6 | 1985 | MeHg |
| Q4QQS8 | Nuclear pore complex protein Nup85 | 58.98 | MeHg |
| P13084 | Nucleophosmin | 287.51 | MeHg |
| Q05982 | Nucleoside diphosphate kinase A | 213.25 | MeHg |
| P19804 | Nucleoside diphosphate kinase B | 352.92 | MeHg |
| P24368 | Peptidyl-prolyl cis-trans isomerase B | 342.4 | MeHg |
| Q63716 | Peroxiredoxin-1 | 184.22 | MeHg |
| P35704 | Peroxiredoxin-2 | 154.09 | MeHg |
| Q9Z0V5 | Peroxiredoxin-4 | 157.54 | MeHg |
| Q9R063 | Peroxiredoxin-5_ mitochondrial | 600.17 | MeHg |
| P31044 | Phosphatidylethanolamine-binding protein 1 | 397.69 | MeHg |
| P16617 | Phosphoglycerate kinase 1 | 159.07 | MeHg |
| P25113 | Phosphoglycerate mutase 1 | 407.48 | MeHg |
| Q9EPH8 | Polyadenylate-binding protein 1 | 79.17 | MeHg |
| Q00438 | Polypyrimidine tract-binding protein 1 | 66.87 | MeHg |
| P0CG51 | Polyubiquitin-B | 576.64 | MeHg |
| Q63429 | Polyubiquitin-C | 576.64 | MeHg |
| P09626 | Potassium-transporting ATPase alpha chain 1 | 26.23 | MeHg |
| P38659 | Protein disulfide-isomerase A4 | 77.64 | MeHg |
| Q5I0H9 | Protein disulfide-isomerase A5 | 60.6 | MeHg |
| O88767 | Protein/nucleic acid deglycase DJ-1 | 295.75 | MeHg |
| P85973 | Purine nucleoside phosphorylase | 96.15 | MeHg |
| P12928 | Pyruvate kinase PKLR | 20.61 | MeHg |
| P11980 | Pyruvate kinase PKM | 99.16 | MeHg |
| P50398 | Rab GDP dissociation inhibitor alpha | 184.21 | MeHg |
| P50399 | Rab GDP dissociation inhibitor beta | 230.7 | MeHg |
| P35281 | Ras-related protein Rab-10 | 400.38 | MeHg |
| P35284 | Ras-related protein Rab-12 | 102.33 | MeHg |
| P35286 | Ras-related protein Rab-13 | 298.05 | MeHg |
| P61107 | Ras-related protein Rab-14 | 102.33 | MeHg |
| P35289 | Ras-related protein Rab-15 | 400.49 | MeHg |
| Q6NYB7 | Ras-related protein Rab-1A | 206.33 | MeHg |
| P10536 | Ras-related protein Rab-1B | 400.38 | MeHg |
| P51156 | Ras-related protein Rab-26 | 102.33 | MeHg |
| Q5U316 | Ras-related protein Rab-35 | 423.02 | MeHg |
| P63012 | Ras-related protein Rab-3A | 102.33 | MeHg |
| Q63941 | Ras-related protein Rab-3B | 102.33 | MeHg |
| P62824 | Ras-related protein Rab-3C | 102.33 | MeHg |
| Q53B90 | Ras-related protein Rab-43 | 102.33 | MeHg |
| P05714 | Ras-related protein Rab-4A | 102.33 | MeHg |
| P51146 | Ras-related protein Rab-4B | 102.33 | MeHg |
| Q9WVB1 | Ras-related protein Rab-6A | 430.91 | MeHg |
| P35280 | Ras-related protein Rab-8A | 400.38 | MeHg |
| P70550 | Ras-related protein Rab-8B | 400.38 | MeHg |
| P63245 | Receptor of activated protein C kinase 1 | 118.87 | MeHg |
| Q5XI73 | Rho GDP-dissociation inhibitor 1 | 245.97 | MeHg |
| P15651 | Short-chain specific acyl-CoA dehydrogenase_ mitochondrial | 80.31 | MeHg |
| P07340 | Sodium/potassium-transporting ATPase subunit beta-1 | 539.03 | MeHg |
| Q66X93 | Staphylococcal nuclease domain-containing protein 1 | 48.12 | MeHg |
| P48721 | Stress-70 protein_ mitochondrial | 42.7 | MeHg |
| Q3MIE4 | Synaptic vesicle membrane protein VAT-1 homolog | 90.13 | MeHg |
| Q5XFX0 | Transgelin-2 | 280.77 | MeHg |
| P46462 | Transitional endoplasmic reticulum ATPase | 198.82 | MeHg |
| Q07984 | Translocon-associated protein subunit delta | 194.36 | MeHg |
| Q5I0E7 | Transmembrane emp24 domain-containing protein 9 | 338.87 | MeHg |
| Q64428 | Trifunctional enzyme subunit alpha_ mitochondrial | 112.18 | MeHg |
| P48500 | Triosephosphate isomerase | 1294.53 | MeHg |
| P62982 | Ubiquitin-40S ribosomal protein S27a | 576.64 | MeHg |
| P62986 | Ubiquitin-60S ribosomal protein L40 | 576.64 | MeHg |
| Q5U300 | Ubiquitin-like modifier-activating enzyme 1 | 83.15 | MeHg |
| P31000 | Vimentin | 59.91 | MeHg |

^a^ Accession ID according to the Uniport.org database.
