# Supplementary material for: Maternal methylmercury exposure changes the proteomic profile of the offspring’s salivary glands: Prospects on translational toxicology
Source: PLoS One. 2021 Nov 8;16(11):e0258969. doi: 10.1371/journal.pone.0258969 (PMC8575261; doi:10.1371/journal.pone.0258969)
Supplement: S4 Table — (DOCX) [file pone.0258969.s004.docx]

**Table S4.** Identified proteins with significantly different expression altered in Parotid Gland of offspring rats of the MeHg group vs. control group.

| Accession ID^a^ | Description | *PLGS* Score | Fold change |
| --- | --- | --- | --- |
| A9UMV8 | Histone H2A.J | 18301.33 | +1.682 |
| P00689 | Pancreatic alpha-amylase | 383.99 | +2.117 |
| P01946 | Hemoglobin subunit alpha-1/2 | 4161.3 | +2.316 |
| P02262 | Histone H2A type 1 | 18301.33 | +1.665 |
| P02770 | Serum albumin | 2161.78 | +1.616 |
| P0C0S7 | Histone H2A.Z | 953.55 | +1.507 |
| P0C169 | Histone H2A type 1-C | 18301.33 | +1.649 |
| P0C170 | Histone H2A type 1-E | 18301.33 | +1.682 |
| P0CC09 | Histone H2A type 2-A | 18301.33 | +1.716 |
| P10719 | ATP synthase subunit beta_ mitochondrial | 73.81 | +1.284 |
| P11517 | Hemoglobin subunit beta-2 | 1011.89 | +1.537 |
| P12020 | Cysteine-rich secretory protein 1 | 1218.98 | +2.014 |
| P21704 | Deoxyribonuclease-1 | 4323.99 | +1.682 |
| P60711 | Actin_ cytoplasmic 1 | 4645.23 | +1.197 |
| P62738 | Actin_ aortic smooth muscle | 583.68 | +1.537 |
| P63039 | 60 kDa heat shock protein_ mitochondrial | 218.48 | +1.600 |
| P63259 | Actin_ cytoplasmic 2 | 4645.23 | +1.185 |
| P63269 | Actin_ gamma-enteric smooth muscle | 583.68 | +1.584 |
| P68035 | Actin_ alpha cardiac muscle | 583.68 | +1.537 |
| P68136 | Actin_ alpha skeletal muscle | 583.68 | +1.553 |
| P84245 | Histone H3.3 | 4501.51 | +1.433 |
| Q00715 | Histone H2B type 1 | 7021.46 | +3.387 |
| Q00728 | Histone H2A type 4 | 18301.33 | +1.616 |
| Q00729 | Histone H2B type 1-A | 435.75 | +2.945 |
| Q4FZT6 | Histone H2A type 3 | 18301.33 | +1.665 |
| Q64598 | Histone H2A type 1-F | 18301.33 | +1.665 |
| Q6RY07 | Acidic mammalian chitinase | 3555.72 | +1.584 |
| P04797 | Glyceraldehyde-3-phosphate dehydrogenase | 1297.14 | +1.336 |
| P46462 | Transitional endoplasmic reticulum ATPase | 137.12 | +1.954 |
| P49242 | 40S ribosomal protein S3a | 48.23 | +2.915 |
| P56574 | Isocitrate dehydrogenase [NADP]_ mitochondrial | 66.89 | –0.698 |
| P19945 | 60S acidic ribosomal protein P0 | 164.16 | –0.522 |
| P17764 | Acetyl-CoA acetyltransferase_ mitochondrial | 328.94 | –0.651 |
| P62083 | 40S ribosomal protein S7 | 2934.09 | –0.779 |
| Q66HD0 | Endoplasmin | 309.22 | –0.771 |
| P11598 | Protein disulfide-isomerase A3 | 522.73 | –0.748 |
| P62963 | Profilin-1 | 4441.14 | –0.638 |
| Q6NYB7 | Ras-related protein Rab-1A | 316.89 | –0.445 |
| P04785 | Protein disulfide-isomerase | 4334.71 | –0.543 |
| P06761 | Endoplasmic reticulum chaperone BiP | 4095.01 | –0.677 |
| P07153 | Dolichyl-diphosphooligosaccharide--protein glycosyltransferase subunit 1 | 208.48 | –0.625 |
| P11240 | Cytochrome c oxidase subunit 5A_ mitochondrial | 659.03 | –0.323 |
| P18418 | Calreticulin | 2095.68 | –0.600 |
| P48037 | Annexin A6 | 187.13 | –0.423 |
| P62890 | 60S ribosomal protein L30 | 3397.13 | –0.383 |
| P69897 | Tubulin beta-5 chain | 1837.82 | –0.698 |
| P85108 | Tubulin beta-2A chain | 1830.2 | –0.677 |
| Q07984 | Translocon-associated protein subunit delta | 589.42 | –0.270 |
| Q3KRE8 | Tubulin beta-2B chain | 1830.2 | –0.677 |
| Q4QRB4 | Tubulin beta-3 chain | 656.31 | –0.712 |
| Q6P9T8 | Tubulin beta-4B chain | 1936.51 | –0.670 |

^a^Accession ID according to the Uniport.org database. Signs of + or – indicate up- or down-regulation, respectively, when MeHg group is compared to control.
