# Supplementary material for: Maternal methylmercury exposure changes the proteomic profile of the offspring’s salivary glands: Prospects on translational toxicology
Source: PLoS One. 2021 Nov 8;16(11):e0258969. doi: 10.1371/journal.pone.0258969 (PMC8575261; doi:10.1371/journal.pone.0258969)
Supplement: S5 Table — (DOCX) [file pone.0258969.s005.docx]

**Table S5.** Identified proteins with significantly different expression altered in submandibular gland of offspring rats MeHg group vs. control group

| Accession ID^a^ | Description | *PLGS*  Score | Fold change |
| --- | --- | --- | --- |
| P00689 | Pancreatic alpha-amylase | 2.386911 | +2.387 |
| P21704 | Deoxyribonuclease-1 | 3.158193 | +3.158 |
| Q4QR99 | Queuine tRNA-ribosyltransferase catalytic subunit 1 | 2.159766 | +2.160 |
| Q68FQ0 | T-complex protein 1 subunit epsilon | 3.781044 | +3.781 |
| O88752 | Epsilon 1 globin | 1.040811 | +1.041 |
| P09626 | Potassium-transporting ATPase alpha chain 1 | 0.657047 | –0.657 |
| P18666 | Myosin regulatory light chain 12B | 0.527292 | 0.527 |
| P50237 | Sulfotransferase 1C1 | 0.763379 | 0.763 |
| P52555 | Endoplasmic reticulum resident protein 29 | 0.650509 | 0.651 |
| P85972 | Vinculin | 0.582748 | 0.583 |
| P70623 | Fatty acid-binding protein_ adipocyte | 0.677057 | 0.677 |
| Q6IFW6 | Keratin_ type I cytoskeletal 10 | 0.501576 | 0.502 |
| P04644 | 40S ribosomal protein S17 | 0.843665 | 0.844 |
| P07335 | Creatine kinase B-type | 0.67032 | 0.670 |
| P12001 | 60S ribosomal protein L18 | 0.548812 | 0.549 |
| P80386 | 5'-AMP-activated protein kinase subunit beta-1 | 0.637628 | 0.638 |
| Q5FVM4 | Non-POU domain-containing octamer-binding protein | 0.486752 | 0.487 |
| Q6IFV4 | Keratin_ type I cytoskeletal 13 | 0.511709 | 0.512 |
| Q9EQS0 | Transaldolase | 0.726149 | 0.726 |
| C9WPN6 | Eukaryotic translation initiation factor 2 subunit 3_ Y-linked | 0.283654 | 0.284 |
| P01026 | Complement C3 | 0.554327 | 0.554 |
| P01946 | Hemoglobin subunit alpha-1/2 | 0.941765 | 0.942 |
| P05371 | Clusterin | 0.571209 | 0.571 |
| P09117 | Fructose-bisphosphate aldolase C | 0.778801 | 0.779 |
| P14046 | Alpha-1-inhibitor 3 | 0.357007 | 0.357 |
| P23965 | Enoyl-CoA delta isomerase 1_ mitochondrial | 0.600496 | 0.600 |
| P47198 | 60S ribosomal protein L22 | 0.740818 | 0.741 |
| P80254 | D-dopachrome decarboxylase | 0.612626 | 0.613 |
| Q03626 | Murinoglobulin-1 | 0.360595 | 0.361 |
| Q3B8Q2 | Eukaryotic initiation factor 4A-III | 0.690734 | 0.691 |
| Q3T1K5 | F-actin-capping protein subunit alpha-2 | 0.625002 | 0.625 |
| Q4KM73 | UMP-CMP kinase | 0.697676 | 0.698 |
| Q5PQJ6 | Pyrroline-5-carboxylate reductase 3 | 0.571209 | 0.571 |
| Q63617 | Hypoxia up-regulated protein 1 | 0.436049 | 0.436 |
| Q6IFX1 | Keratin_ type I cytoskeletal 24 | 0.472367 | 0.472 |
| Q6P799 | Serine--tRNA ligase_ cytoplasmic | 0.458406 | 0.458 |
| A9UMV8 | Histone H2A.J | 0.440432 | 0.440 |
| B2GV06 | Succinyl-CoA:3-ketoacid coenzyme A transferase 1_ mitochondrial | 0.606531 | 0.607 |
| B4F7E8 | Niban-like protein 1 | 0.410656 | 0.411 |
| G3V7G8 | Glycine--tRNA ligase | 0.559898 | 0.560 |
| O35763 | Moesin | 0.386741 | 0.387 |
| O35854 | Branched-chain-amino-acid aminotransferase_ mitochondrial | 0.548812 | 0.549 |
| O70351 | 3-hydroxyacyl-CoA dehydrogenase type-2 | 0.582748 | 0.583 |
| O70417 | Prolactin-inducible protein homolog | 0.386741 | 0.387 |
| O88767 | Protein/nucleic acid deglycase DJ-1 | 0.57695 | 0.577 |
| O88989 | Malate dehydrogenase_ cytoplasmic | 0.571209 | 0.571 |
| P00406 | Cytochrome c oxidase subunit 2 | 0.594521 | 0.595 |
| P00507 | Aspartate aminotransferase_ mitochondrial | 0.506617 | 0.507 |
| P00758 | Kallikrein-1 | 0.398519 | 0.399 |
| P02262 | Histone H2A type 1 | 0.644036 | 0.644 |
| P02401 | 60S acidic ribosomal protein P2 | 0.349938 | 0.350 |
| P02454 | Collagen alpha-1(I) chain | 0.516851 | 0.517 |
| P02770 | Serum albumin | 0.527292 | 0.527 |
| P04182 | Ornithine aminotransferase_ mitochondrial | 0.843665 | 0.844 |
| P04636 | Malate dehydrogenase_ mitochondrial | 0.532592 | 0.533 |
| P04642 | L-lactate dehydrogenase A chain | 0.631284 | 0.631 |
| P04762 | Catalase | 0.444858 | 0.445 |
| P04764 | Alpha-enolase | 0.414783 | 0.415 |
| P04785 | Protein disulfide-isomerase | 0.336216 | 0.336 |
| P04797 | Glyceraldehyde-3-phosphate dehydrogenase | 0.625002 | 0.625 |
| P04906 | Glutathione S-transferase P | 0.486752 | 0.487 |
| P05065 | Fructose-bisphosphate aldolase A | 0.67032 | 0.670 |
| P05197 | Elongation factor 2 | 0.453845 | 0.454 |
| P05714 | Ras-related protein Rab-4A | 0.390628 | 0.391 |
| P06685 | Sodium/potassium-transporting ATPase subunit alpha-1 | 0.501576 | 0.502 |
| P06686 | Sodium/potassium-transporting ATPase subunit alpha-2 | 0.527292 | 0.527 |
| P06687 | Sodium/potassium-transporting ATPase subunit alpha-3 | 0.516851 | 0.517 |
| P06761 | Endoplasmic reticulum chaperone BiP | 0.472367 | 0.472 |
| P07153 | Dolichyl-diphosphooligosaccharide--protein glycosyltransferase subunit 1 | 0.600496 | 0.600 |
| P07323 | Gamma-enolase | 0.458406 | 0.458 |
| P07340 | Sodium/potassium-transporting ATPase subunit beta-1 | 0.449329 | 0.449 |
| P07633 | Propionyl-CoA carboxylase beta chain_ mitochondrial | 0.71177 | 0.712 |
| P08009 | Glutathione S-transferase Yb-3 | 0.19398 | 0.194 |
| P08010 | Glutathione S-transferase Mu 2 | 0.527292 | 0.527 |
| P08461 | Dihydrolipoyllysine-residue acetyltransferase component of pyruvate dehydrogenase complex_ mitochondrial | 0.444858 | 0.445 |
| P08462 | Submandibular gland secretory Glx-rich protein CB | 0.22091 | 0.221 |
| P09606 | Glutamine synthetase | 0.733447 | 0.733 |
| P09895 | 60S ribosomal protein L5 | 0.472367 | 0.472 |
| P0C0S7 | Histone H2A.Z | 0.463013 | 0.463 |
| P0C169 | Histone H2A type 1-C | 0.436049 | 0.436 |
| P0C170 | Histone H2A type 1-E | 0.644036 | 0.644 |
| P0CC09 | Histone H2A type 2-A | 0.436049 | 0.436 |
| P0DMW0 | Heat shock 70 kDa protein 1A | 0.600496 | 0.600 |
| P0DMW1 | Heat shock 70 kDa protein 1B | 0.594521 | 0.595 |
| P0DP29 | Calmodulin-1 | 0.548812 | 0.549 |
| P0DP30 | Calmodulin-2 | 0.559898 | 0.560 |
| P0DP31 | Calmodulin-3 | 0.559898 | 0.560 |
| P10111 | Peptidyl-prolyl cis-trans isomerase A | 0.588605 | 0.589 |
| P10536 | Ras-related protein Rab-1B | 0.481909 | 0.482 |
| P10719 | ATP synthase subunit beta_ mitochondrial | 0.522046 | 0.522 |
| P10760 | Adenosylhomocysteinase | 0.690734 | 0.691 |
| P11030 | Acyl-CoA-binding protein | 0.463013 | 0.463 |
| P11240 | Cytochrome c oxidase subunit 5A_ mitochondrial | 0.606531 | 0.607 |
| P11442 | Clathrin heavy chain 1 | 0.57695 | 0.577 |
| P11598 | Protein disulfide-isomerase A3 | 0.491644 | 0.492 |
| P11884 | Aldehyde dehydrogenase_ mitochondrial | 0.332871 | 0.333 |
| P11980 | Pyruvate kinase PKM | 0.537944 | 0.538 |
| P12346 | Serotransferrin | 0.522046 | 0.522 |
| P12928 | Pyruvate kinase PKLR | 0.423162 | 0.423 |
| P13084 | Nucleophosmin | 0.690734 | 0.691 |
| P13221 | Aspartate aminotransferase_ cytoplasmic | 0.554327 | 0.554 |
| P13601 | Aldehyde dehydrogenase_ cytosolic 1 | 0.491644 | 0.492 |
| P13803 | Electron transfer flavoprotein subunit alpha_ mitochondrial | 0.565525 | 0.566 |
| P14408 | Fumarate hydratase_ mitochondrial | 0.506617 | 0.507 |
| P14604 | Enoyl-CoA hydratase_ mitochondrial | 0.537944 | 0.538 |
| P14659 | Heat shock-related 70 kDa protein 2 | 0.606531 | 0.607 |
| P15429 | Beta-enolase | 0.501576 | 0.502 |
| P15999 | ATP synthase subunit alpha_ mitochondrial | 0.444858 | 0.445 |
| P16617 | Phosphoglycerate kinase 1 | 0.677057 | 0.677 |
| P16638 | ATP-citrate synthase | 0.449329 | 0.449 |
| P17077 | 60S ribosomal protein L9 | 0.496585 | 0.497 |
| P17764 | Acetyl-CoA acetyltransferase_ mitochondrial | 0.697676 | 0.698 |
| P18418 | Calreticulin | 0.527292 | 0.527 |
| P18596 | Sarcoplasmic/endoplasmic reticulum calcium ATPase 3 | 0.511709 | 0.512 |
| P19804 | Nucleoside diphosphate kinase B | 0.516851 | 0.517 |
| P19944 | 60S acidic ribosomal protein P1 | 0.631284 | 0.631 |
| P19945 | 60S acidic ribosomal protein P0 | 0.481909 | 0.482 |
| P20280 | 60S ribosomal protein L21 | 0.496585 | 0.497 |
| P20761 | Ig gamma-2B chain C region | 0.594521 | 0.595 |
| P20788 | Cytochrome b-c1 complex subunit Rieske_ mitochondrial | 0.522046 | 0.522 |
| P21531 | 60S ribosomal protein L3 | 0.467666 | 0.468 |
| P21913 | Succinate dehydrogenase [ubiquinone] iron-sulfur subunit_ mitochondrial | 0.565525 | 0.566 |
| P23514 | Coatomer subunit beta | 0.349938 | 0.350 |
| P24090 | Alpha-2-HS-glycoprotein | 0.618783 | 0.619 |
| P24268 | Cathepsin D | 0.588605 | 0.589 |
| P24368 | Peptidyl-prolyl cis-trans isomerase B | 0.40657 | 0.407 |
| P25113 | Phosphoglycerate mutase 1 | 0.543351 | 0.543 |
| P25809 | Creatine kinase U-type_ mitochondrial | 0.612626 | 0.613 |
| P27139 | Carbonic anhydrase 2 | 0.394554 | 0.395 |
| P27952 | 40S ribosomal protein S2 | 0.467666 | 0.468 |
| P29147 | D-beta-hydroxybutyrate dehydrogenase_ mitochondrial | 0.382893 | 0.383 |
| P29266 | 3-hydroxyisobutyrate dehydrogenase_ mitochondrial | 0.571209 | 0.571 |
| P30904 | Macrophage migration inhibitory factor | 0.657047 | 0.657 |
| P31000 | Vimentin OS=Rattus norvegicus | 0.527292 | 0.527 |
| P31044 | Phosphatidylethanolamine-binding protein 1 | 0.618783 | 0.619 |
| P31977 | Ezrin | 0.427415 | 0.427 |
| P32551 | Cytochrome b-c1 complex subunit 2_ mitochondrial | 0.463013 | 0.463 |
| P34058 | Heat shock protein HSP 90-beta | 0.375311 | 0.375 |
| P35213 | 14-3-3 protein beta/alpha | 0.357007 | 0.357 |
| P35280 | Ras-related protein Rab-8A | 0.472367 | 0.472 |
| P35281 | Ras-related protein Rab-10 | 0.481909 | 0.482 |
| P35284 | Ras-related protein Rab-12 | 0.390628 | 0.391 |
| P35286 | Ras-related protein Rab-13 | 0.644036 | 0.644 |
| P35289 | Ras-related protein Rab-15 | 0.491644 | 0.492 |
| P35704 | Peroxiredoxin-2 | 0.527292 | 0.527 |
| P36972 | Adenine phosphoribosyltransferase | 0.554327 | 0.554 |
| P38983 | 40S ribosomal protein AS | 0.57695 | 0.577 |
| P42123 | L-lactate dehydrogenase B chain | 0.316637 | 0.317 |
| P45592 | Cofilin-1 | 0.726149 | 0.726 |
| P46462 | Transitional endoplasmic reticulum ATPase | 0.516851 | 0.517 |
| P48037 | Annexin A6 | 0.582748 | 0.583 |
| P48500 | Triosephosphate isomerase | 0.532592 | 0.533 |
| P48679 | Prelamin-A/C | 0.631284 | 0.631 |
| P48721 | Stress-70 protein_ mitochondrial | 0.637628 | 0.638 |
| P49088 | Asparagine synthetase [glutamine-hydrolyzing] | 0.486752 | 0.487 |
| P49242 | 40S ribosomal protein S3a | 0.565525 | 0.566 |
| P49432 | Pyruvate dehydrogenase E1 component subunit beta_ mitochondrial | 0.600496 | 0.600 |
| P50137 | Transketolase | 0.625002 | 0.625 |
| P50398 | Rab GDP dissociation inhibitor alpha | 0.486752 | 0.487 |
| P50399 | Rab GDP dissociation inhibitor beta | 0.537944 | 0.538 |
| P51146 | Ras-related protein Rab-4B | 0.390628 | 0.391 |
| P51156 | Ras-related protein Rab-26 | 0.386741 | 0.387 |
| P51647 | Retinal dehydrogenase 1 | 0.516851 | 0.517 |
| P51657 | Estradiol 17-beta-dehydrogenase 1 | 0.565525 | 0.566 |
| P52303 | AP-1 complex subunit beta-1 | 0.481909 | 0.482 |
| P52873 | Pyruvate carboxylase_ mitochondrial | 0.516851 | 0.517 |
| P54708 | Potassium-transporting ATPase alpha chain 2 | 0.606531 | 0.487 |
| P55063 | Heat shock 70 kDa protein 1-like | 0.516851 | 0.607 |
| P56574 | Isocitrate dehydrogenase [NADP]_ mitochondrial | 0.516851 | 0.517 |
| P60711 | Actin_ cytoplasmic 1 | 0.600496 | 0.517 |
| P61107 | Ras-related protein Rab-14 | 0.390628 | 0.600 |
| P61206 | ADP-ribosylation factor 3 | 0.382893 | 0.391 |
| P61751 | ADP-ribosylation factor 4 | 0.431711 | 0.383 |
| P61980 | Heterogeneous nuclear ribonucleoprotein K | 0.650509 | 0.432 |
| P61983 | 14-3-3 protein gamma | 0.382893 | 0.651 |
| P62083 | 40S ribosomal protein S7 | 0.588605 | 0.383 |
| P62243 | 40S ribosomal protein S8 | 0.582748 | 0.589 |
| P62246 | 40S ribosomal protein S15a | 0.414783 | 0.583 |
| P62250 | 40S ribosomal protein S16 | 0.458406 | 0.415 |
| P62260 | 14-3-3 protein epsilon | 0.26982 | 0.458 |
| P62630 | Elongation factor 1-alpha 1 | 0.594521 | 0.270 |
| P62632 | Elongation factor 1-alpha 2 | 0.477114 | 0.595 |
| P62738 | Actin_ aortic smooth muscle | 0.582748 | 0.477 |
| P62804 | Histone H4 | 0.472367 | 0.583 |
| P62824 | Ras-related protein Rab-3C | 0.386741 | 0.472 |
| P62845 | 40S ribosomal protein S15 | 0.227638 | 0.387 |
| P62890 | 60S ribosomal protein L30 | 0.612626 | 0.228 |
| P62898 | Cytochrome c_ somatic | 0.316637 | 0.613 |
| P62909 | 40S ribosomal protein S3 | 0.390628 | 0.317 |
| P62914 | 60S ribosomal protein L11 | 0.410656 | 0.391 |
| P62963 | Profilin-1 | 0.71177 | 0.411 |
| P63012 | Ras-related protein Rab-3A | 0.382893 | 0.712 |
| P63018 | Heat shock cognate 71 kDa protein | 0.537944 | 0.383 |
| P63029 | Translationally-controlled tumor protein | 0.382893 | 0.538 |
| P63039 | 60 kDa heat shock protein_ mitochondrial | 0.582748 | 0.383 |
| P63102 | 14-3-3 protein zeta/delta | 0.477114 | 0.583 |
| P63245 | Receptor of activated protein C kinase 1 | 0.394554 | 0.477 |
| P63259 | Actin_ cytoplasmic 2 | 0.612626 | 0.395 |
| P63269 | Actin_ gamma-enteric smooth muscle | 0.582748 | 0.613 |
| P67779 | Prohibitin | 0.657047 | 0.583 |
| P68035 | Actin_ alpha cardiac muscle 1 | 0.57695 | 0.657 |
| P68136 | Actin_ alpha skeletal muscle | 0.588605 | 0.577 |
| P68255 | 14-3-3 protein theta | 0.29523 | 0.589 |
| P68370 | Tubulin alpha-1A chain | 0.57695 | 0.295 |
| P68511 | 14-3-3 protein eta | 0.343008 | 0.577 |
| P69897 | Tubulin beta-5 chain | 0.496585 | 0.343 |
| P70550 | Ras-related protein Rab-8B | 0.467666 | 0.497 |
| P81155 | Voltage-dependent anion-selective channel protein 2 | 0.588605 | 0.468 |
| P81795 | Eukaryotic translation initiation factor 2 subunit 3_ X-linked | 0.588605 | 0.589 |
| P82808 | Glutamine--fructose-6-phosphate aminotransferase [isomerizing] 1 | 0.349938 | 0.589 |
| P82995 | Heat shock protein HSP 90-alpha | 0.357007 | 0.350 |
| P84079 | ADP-ribosylation factor 1 | 0.379083 | 0.357 |
| P84082 | ADP-ribosylation factor 2 | 0.427415 | 0.379 |
| P84083 | ADP-ribosylation factor 5 | 0.440432 | 0.427 |
| P84245 | Histone H3.3 | 0.444858 | 0.440 |
| P85108 | Tubulin beta-2A chain | 0.491644 | 0.445 |
| P85973 | Purine nucleoside phosphorylase | 0.650509 | 0.492 |
| Q00438 | Polypyrimidine tract-binding protein 1 | 0.427415 | 0.651 |
| Q00715 | Histone H2B type 1 | 0.423162 | 0.427 |
| Q00728 | Histone H2A type 4 | 0.440432 | 0.423 |
| Q00729 | Histone H2B type 1-A | 0.472367 | 0.440 |
| Q02253 | Methylmalonate-semialdehyde dehydrogenase [acylating]_ mitochondrial | 0.704688 | 0.472 |
| Q05962 | ADP/ATP translocase 1 | 0.582748 | 0.705 |
| Q05982 | Nucleoside diphosphate kinase A | 0.501576 | 0.583 |
| Q06647 | ATP synthase subunit O_ mitochondrial | 0.386741 | 0.502 |
| Q07936 | Annexin A2 | 0.543351 | 0.387 |
| Q07984 | Translocon-associated protein subunit delta | 0.486752 | 0.543 |
| Q08163 | Adenylyl cyclase-associated protein 1 | 0.477114 | 0.487 |
| Q09073 | ADP/ATP translocase 2 | 0.571209 | 0.477 |
| Q10758 | Keratin_ type II cytoskeletal 8 | 0.57695 | 0.571 |
| Q3KRE8 | Tubulin beta-2B chain | 0.486752 | 0.577 |
| Q3MIE4 | Synaptic vesicle membrane protein VAT-1 homolog | 0.690734 | 0.487 |
| Q4AEF8 | Coatomer subunit gamma-1 | 0.594521 | 0.691 |
| Q4FZT6 | Histone H2A type 3 | 0.440432 | 0.595 |
| Q4FZU2 | Keratin_ type II cytoskeletal 6A | 0.527292 | 0.440 |
| Q4QRB4 | Tubulin beta-3 chain | 0.501576 | 0.527 |
| Q53B90 | Ras-related protein Rab-43 | 0.431711 | 0.502 |
| Q561S0 | NADH dehydrogenase [ubiquinone] 1 alpha subcomplex subunit 10_ mitochondrial | 0.600496 | 0.432 |
| Q5BJY9 | Keratin_ type I cytoskeletal 18 | 0.532592 | 0.600 |
| Q5I0E7 | Transmembrane emp24 domain-containing protein 9 | 0.537944 | 0.533 |
| Q5RKI1 | Eukaryotic initiation factor 4A-II | 0.71177 | 0.538 |
| Q5U2Q3 | Ester hydrolase C11orf54 homolog | 0.532592 | 0.712 |
| Q5U300 | Ubiquitin-like modifier-activating enzyme 1 | 0.683861 | 0.533 |
| Q5U316 | Ras-related protein Rab-35 | 0.491644 | 0.684 |
| Q5XFX0 | Transgelin-2 | 0.319819 | 0.492 |
| Q5XHZ0 | Heat shock protein 75 kDa_ mitochondrial | 0.346456 | 0.320 |
| Q5XI73 | Rho GDP-dissociation inhibitor 1 | 0.683861 | 0.346 |
| Q5XI78 | 2-oxoglutarate dehydrogenase_ mitochondrial | 0.414783 | 0.684 |
| Q5XIF6 | Tubulin alpha-4A chain | 0.543351 | 0.415 |
| Q5XIH7 | Prohibitin-2 | 0.511709 | 0.543 |
| Q60587 | Trifunctional enzyme subunit beta_ mitochondrial | 0.677057 | 0.512 |
| Q62667 | Major vault protein | 0.740818 | 0.677 |
| Q62812 | Myosin-9 | 0.612626 | 0.741 |
| Q62902 | Protein ERGIC-53 | 0.477114 | 0.613 |
| Q63041 | Alpha-1-macroglobulin | 0.778801 | 0.477 |
| Q63081 | Protein disulfide-isomerase A6 | 0.571209 | 0.779 |
| Q63270 | Cytoplasmic aconitate hydratase | 0.440432 | 0.571 |
| Q63279 | Keratin_ type I cytoskeletal 19 | 0.600496 | 0.440 |
| Q63716 | Peroxiredoxin-1 | 0.582748 | 0.600 |
| Q63941 | Ras-related protein Rab-3B | 0.386741 | 0.583 |
| Q63942 | GTP-binding protein Rab-3D | 0.398519 | 0.387 |
| Q64057 | Alpha-aminoadipic semialdehyde dehydrogenase | 0.726149 | 0.399 |
| Q64119 | Myosin light polypeptide 6 | 0.618783 | 0.726 |
| Q641Y0 | Dolichyl-diphosphooligosaccharide--protein glycosyltransferase 48 kDa subunit | 0.496585 | 0.619 |
| Q64428 | Trifunctional enzyme subunit alpha_ mitochondrial | 0.704688 | 0.497 |
| Q64541 | Sodium/potassium-transporting ATPase subunit alpha-4 | 0.449329 | 0.705 |
| Q64591 | 2_4-dienoyl-CoA reductase_ mitochondrial | 0.677057 | 0.449 |
| Q64598 | Histone H2A type 1-F | 0.440432 | 0.677 |
| Q66H61 | Glutamine--tRNA ligase | 0.481909 | 0.440 |
| Q66HD0 | Endoplasmin | 0.501576 | 0.482 |
| Q66HF1 | NADH-ubiquinone oxidoreductase 75 kDa subunit_ mitochondrial | 0.301194 | 0.502 |
| Q66X93 | Staphylococcal nuclease domain-containing protein 1 | 0.506617 | 0.301 |
| Q68FR6 | Elongation factor 1-gamma | 0.612626 | 0.507 |
| Q68FR8 | Tubulin alpha-3 chain | 0.594521 | 0.613 |
| Q68FR9 | Elongation factor 1-delta | 0.625002 | 0.595 |
| Q68FU3 | Electron transfer flavoprotein subunit beta | 0.740818 | 0.625 |
| Q68FY0 | Cytochrome b-c1 complex subunit 1_ mitochondrial | 0.631284 | 0.741 |
| Q6AY56 | Tubulin alpha-8 chain | 0.588605 | 0.631 |
| Q6AYZ1 | Tubulin alpha-1C chain | 0.594521 | 0.589 |
| Q6IE52 | Murinoglobulin-2 | 0.427415 | 0.595 |
| Q6IFU7 | Keratin_ type I cytoskeletal 42 | 0.582748 | 0.427 |
| Q6IFU8 | Keratin_ type I cytoskeletal 17 | 0.565525 | 0.583 |
| Q6IFV1 | Keratin_ type I cytoskeletal 14 | 0.565525 | 0.566 |
| Q6IFV3 | Keratin_ type I cytoskeletal 15 | 0.565525 | 0.566 |
| Q6IFW2 | Keratin_ type I cytoskeletal 40 | 0.449329 | 0.566 |
| Q6IFW5 | Keratin_ type I cytoskeletal 12 | 0.565525 | 0.449 |
| Q6IG05 | Keratin_ type II cytoskeletal 75 | 0.390628 | 0.566 |
| Q6IG12 | Keratin_ type II cytoskeletal 7 | 0.506617 | 0.391 |
| Q6IMY8 | Heterogeneous nuclear ribonucleoprotein U | 0.612626 | 0.507 |
| Q6LED0 | Histone H3.1 | 0.410656 | 0.613 |
| Q6NYB7 | Ras-related protein Rab-1A | 0.543351 | 0.411 |
| Q6P502 | T-complex protein 1 subunit gamma | 0.637628 | 0.543 |
| Q6P6Q2 | Keratin_ type II cytoskeletal 5 | 0.458406 | 0.638 |
| Q6P6R2 | Dihydrolipoyl dehydrogenase_ mitochondrial | 0.481909 | 0.458 |
| Q6P6V0 | Glucose-6-phosphate isomerase | 0.394554 | 0.482 |
| Q6P9T8 | Tubulin beta-4B chain | 0.565525 | 0.395 |
| Q6P9V9 | Tubulin alpha-1B chain | 0.565525 | 0.566 |
| Q6PDV7 | 60S ribosomal protein L10 | 0.554327 | 0.566 |
| Q794E4 | Heterogeneous nuclear ribonucleoprotein F | 0.650509 | 0.554 |
| Q7M0E3 | Destrin OS=Rattus norvegicus | 0.491644 | 0.651 |
| Q8VHF5 | Citrate synthase_ mitochondrial | 0.57695 | 0.492 |
| Q920L2 | Succinate dehydrogenase [ubiquinone] flavoprotein subunit_ mitochondrial | 0.657047 | 0.577 |
| Q9EPH8 | Polyadenylate-binding protein 1 | 0.650509 | 0.657 |
| Q9ER34 | Aconitate hydratase_ mitochondrial | 0.511709 | 0.651 |
| Q9QX79 | Fetuin-B | 0.467666 | 0.512 |
| Q9QXQ0 | Alpha-actinin-4 | 0.67032 | 0.468 |
| Q9R063 | Peroxiredoxin-5_ mitochondrial | 0.543351 | 0.670 |
| Q9R1Z0 | Voltage-dependent anion-selective channel protein 3 | 0.57695 | 0.543 |
| Q9WVB1 | Ras-related protein Rab-6A | 0.390628 | 0.577 |
| Q9Z0V5 | Peroxiredoxin-4 | 0.449329 | 0.391 |
| Q9Z1A6 | Vigilin | 0.559898 | 0.449 |
| Q9Z1P2 | Alpha-actinin-1 | 0.718924 | 0.560 |
| Q9Z2L0 | Voltage-dependent anion-selective channel protein 1 | 0.496585 | 0.719 |
| Q9Z2Q1 | Protein transport protein Sec31A | 0.57695 | 0.497 |

^a^Accession ID according to the Uniport.org database. Signs of + or – indicate up- or down-regulation, respectively, when MeHg group is compared to control.
