# Supplementary material for: Maternal methylmercury exposure changes the proteomic profile of the offspring’s salivary glands: Prospects on translational toxicology
Source: PLoS One. 2021 Nov 8;16(11):e0258969. doi: 10.1371/journal.pone.0258969 (PMC8575261; doi:10.1371/journal.pone.0258969)
Supplement: S6 Table — (DOCX) [file pone.0258969.s006.docx]

**Table S6.** Identified proteins with significantly different expression altered in sublingual gland of offspring rats of the MeHg group vs. control group

| Accession ID^a^ | Description | PLGS Score | Fold change |
| --- | --- | --- | --- |
| P62083 | 40S ribosomal protein S7 | 320 | +1 |
| P19944 | 60S acidic ribosomal protein P1 | 1322 | +1 |
| P68035 | Actin_ alpha cardiac muscle 1 | 485 | +1 |
| P68136 | Actin_ alpha skeletal muscle | 485 | +1 |
| P62738 | Actin_ aortic smooth muscle | 483 | +1 |
| P60711 | Actin_ cytoplasmic 1 | 1109 | +1 |
| P63259 | Actin_ cytoplasmic 2 | 1109 | +1 |
| P63269 | Actin_ gamma-enteric smooth muscle | 483 | +1 |
| P15999 | ATP synthase subunit alpha_ mitochondrial | 108 | +1 |
| P10719 | ATP synthase subunit beta_ mitochondrial | 716 | +1 |
| P18418 | Calreticulin | 475 | +1 |
| P11240 | Cytochrome c oxidase subunit 5A_ mitochondrial | 160 | +1 |
| P62630 | Elongation factor 1-alpha 1 | 429 | +1 |
| P62632 | Elongation factor 1-alpha 2 | 19 | +1 |
| Q66HD0 | Endoplasmin | 136 | +1 |
| P04797 | Glyceraldehyde-3-phosphate dehydrogenase | 264 | +1 |
| P01946 | Hemoglobin subunit alpha-1/2 | 2502 | +1 |
| P02091 | Hemoglobin subunit beta-1 | 1224 | +1 |
| P11517 | Hemoglobin subunit beta-2 | 362 | +1 |
| P02262 | Histone H2A type 1 | 6379 | +1 |
| P0C169 | Histone H2A type 1-C | 6379 | +1 |
| P0C170 | Histone H2A type 1-E | 6379 | +1 |
| Q64598 | Histone H2A type 1-F | 6379 | +1 |
| P0CC09 | Histone H2A type 2-A | 6379 | +1 |
| Q4FZT6 | Histone H2A type 3 | 6379 | +1 |
| Q00728 | Histone H2A type 4 | 6379 | +1 |
| A9UMV8 | Histone H2A.J | 6379 | +1 |
| P0C0S7 | Histone H2A.Z | 1072 | +1 |
| Q00715 | Histone H2B type 1 | 395 | +1 |
| Q00729 | Histone H2B type 1-A | 193 | +1 |
| P62804 | Histone H4 | 1701 | +1 |
| Q5BJY9 | Keratin_ type I cytoskeletal 18 | 73 | +1 |
| Q63279 | Keratin_ type I cytoskeletal 19 | 46 | +1 |
| Q10758 | Keratin_ type II cytoskeletal 8 | 355 | +1 |
| O88989 | Malate dehydrogenase_ cytoplasmic | 97 | +1 |
| P00689 | Pancreatic alpha-amylase | 105 | +1 |
| P10111 | Peptidyl-prolyl cis-trans isomerase A | 428 | +1 |
| P62963 | Profilin-1 | 734 | +1 |
| P11598 | Protein disulfide-isomerase A3 | 196 | +1 |
| P04785 | Protein disulfide-isomerase | 140 | +1 |
| P12346 | Serotransferrin | 62 | +1 |
| P02770 | Serum albumin | 384 | +1 |
| P06685 | Sodium/potassium-transporting ATPase subunit alpha-1 | 50 | +1 |
| P68370 | Tubulin alpha-1A chain | 134 | +1 |
| Q6P9V9 | Tubulin alpha-1B chain | 134 | +1 |
| Q6AYZ1 | Tubulin alpha-1C chain | 140 | +1 |
| Q4QRB4 | Tubulin beta-3 chain | 284 | +1 |
| Q6P9T8 | Tubulin beta-4B chain | 679 | +1 |
| P06686 | Sodium/potassium-transporting ATPase subunit alpha-2 | 31 | –0.99 |
| P06687 | Sodium/potassium-transporting ATPase subunit alpha-3 | 31 | –0.99 |
| P85108 | Tubulin beta-2A chain | 600 | –0.98 |
| Q3KRE8 | Tubulin beta-2B chain | 600 | –0.98 |
| P69897 | Tubulin beta-5 chain | 581 | –0.98 |
| Q3B8Q2 | Eukaryotic initiation factor 4A-III | 113 | –0.02 |

^a^Accession ID according to the Uniport.org database. Signs of + or – indicate up- or down-regulation, respectively, when MeHg group is compared to control.
