# Supplementary material for: Maternal methylmercury exposure changes the proteomic profile of the offspring’s salivary glands: Prospects on translational toxicology
Source: PLoS One. 2021 Nov 8;16(11):e0258969. doi: 10.1371/journal.pone.0258969 (PMC8575261; doi:10.1371/journal.pone.0258969)

**Table S7.** The ARRIVE Guidelines Checklist


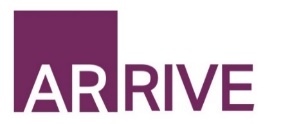


Animal Research: Reporting In Vivo Experiments

Carol Kilkenny^1^, William J Browne^2^, Innes C Cuthill^3^, Michael Emerson^4^ and Douglas G Altman^5^

*^1^The National Centre for the Replacement, Refinement and Reduction of Animals in Research, London, UK, ^2^School of Veterinary Science, University of Bristol, Bristol, UK, ^3^School of Biological Sciences, University of Bristol, Bristol, UK, ^4^National Heart and Lung Institute, Imperial College London, UK, ^5^Centre for Statistics in Medicine, University of Oxford, Oxford, UK.*

|  | | ITEM | RECOMMENDATION | Section/ Paragraph |
| --- | --- | --- | --- | --- |
| 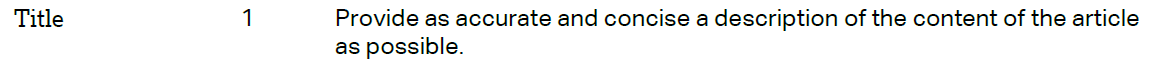 | | | Title page |  |
| 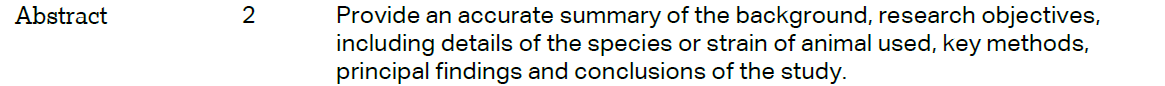 | | | Abstract section |  |
| INTRODUCTION | | |  |  |
| 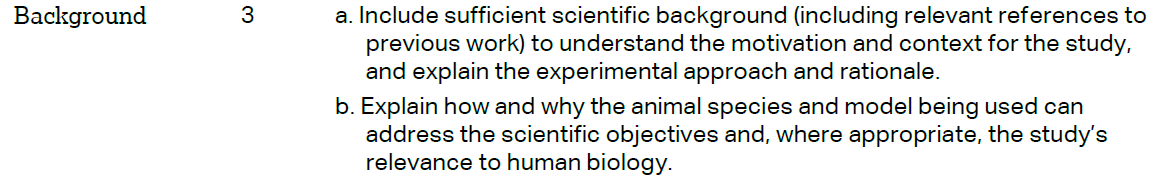 | | | Abstract section; Introduction |  |
| 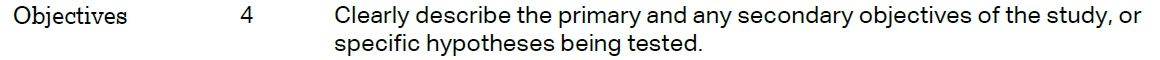 | | | Introduction (last paragraph) |  |
| METHODS | | |  |  |
| 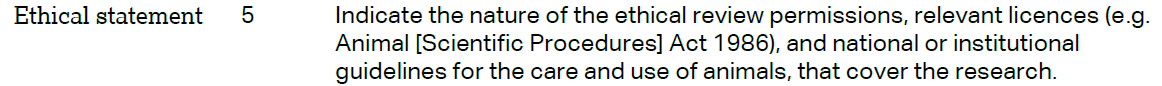 | | | 1^st^ paragraph MM |  |
| 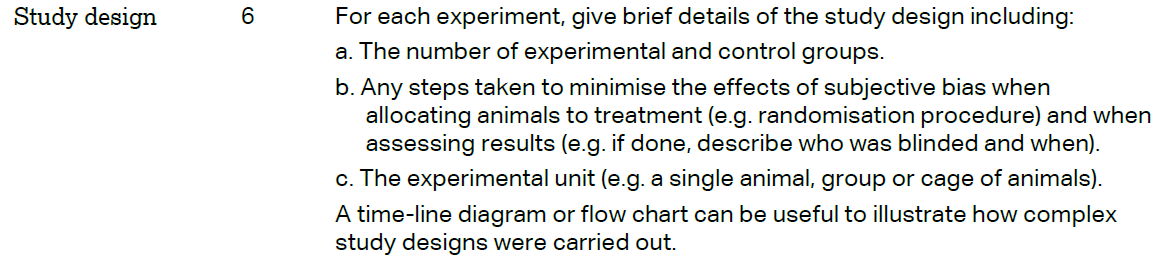 | | | 1^st^ section MM |  |
| 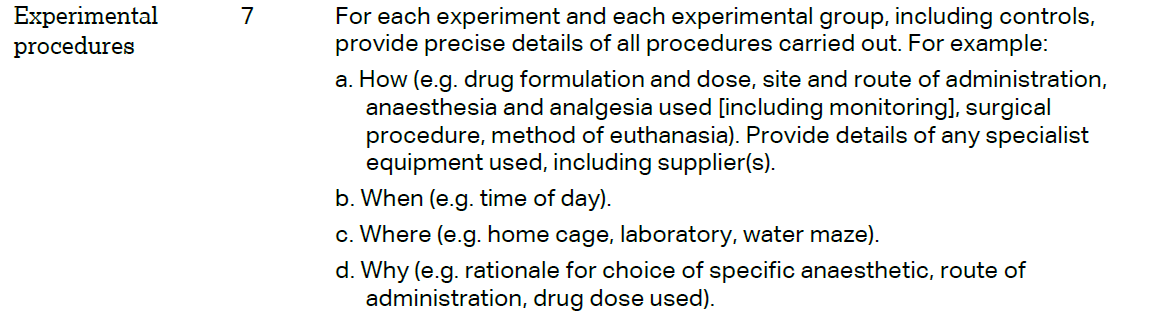 | | | 2^nd^ section MM |  |
| 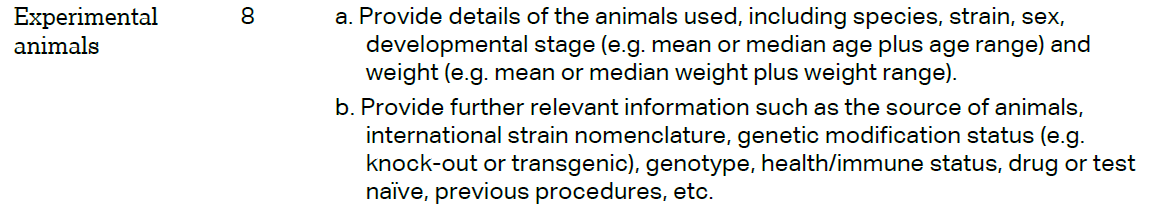 | | | 1^st^ section MM |  |

The ARRIVE guidelines. Originally published in *PLoS Biology*, June 2010^1^

| 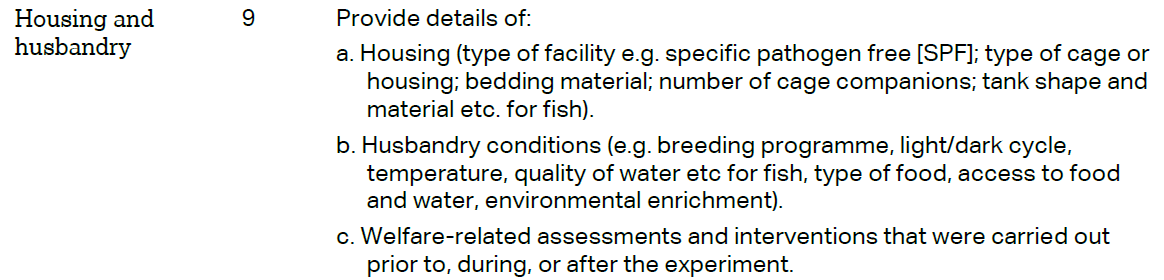 | 1^st^ section MM | |
| --- | --- | --- |
| 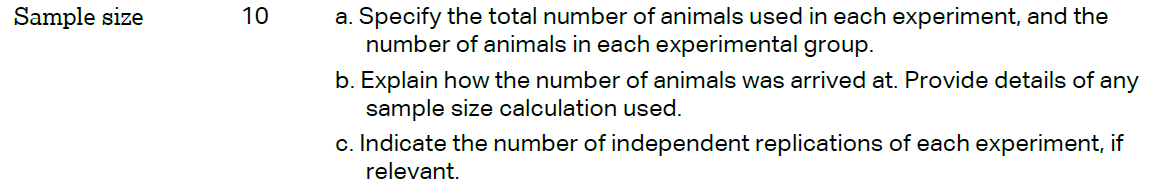 | 1^st^ section MM | |
| 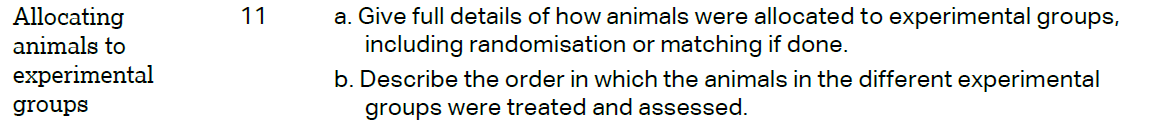 | Fig.1. | |
| 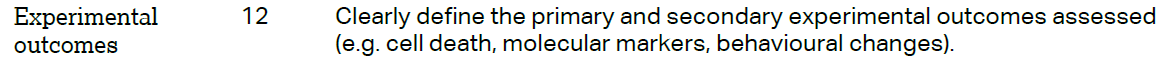 | Fig.1. | |
| 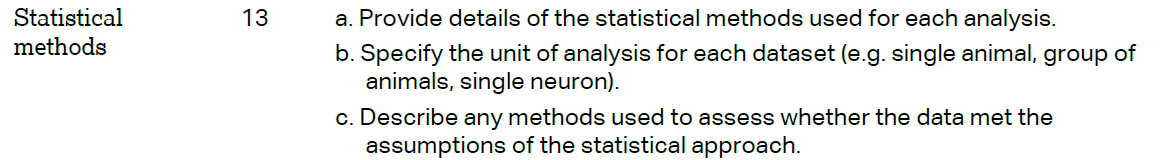 | Last section MM | |
| RESULTS |  | |
| 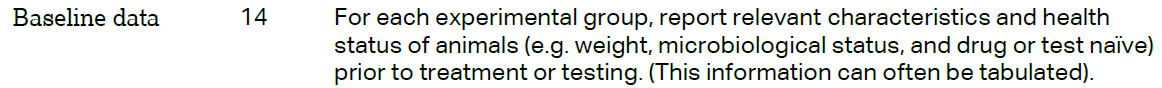 | Table Supplementary in Nascimento et al 2020 [12] | |
| 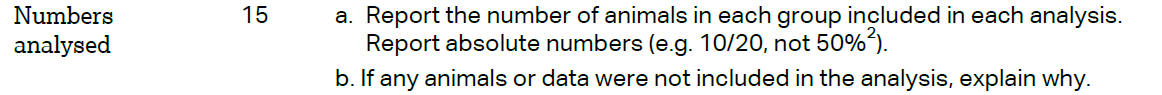 | 1^st^ section | |
| 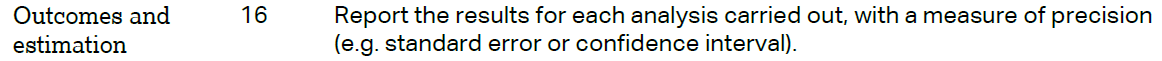 | 2^nd^ section | |
| 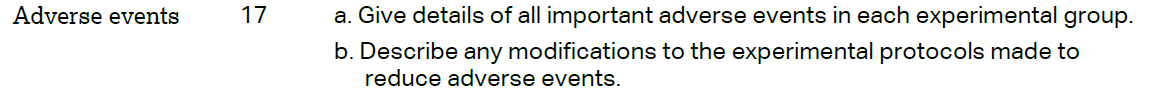 | 2^nd^ section | |
| DISCUSSION |  | |
| 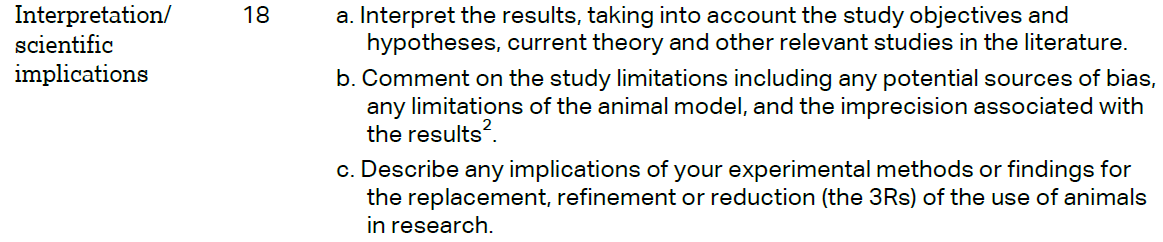 | 17-23p | |
| 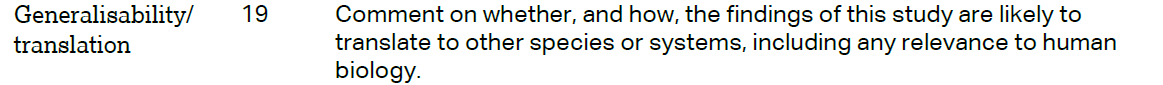 | Last paragraph | |
| 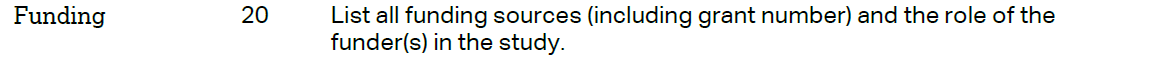 | | Funding Statement |


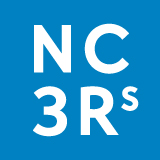

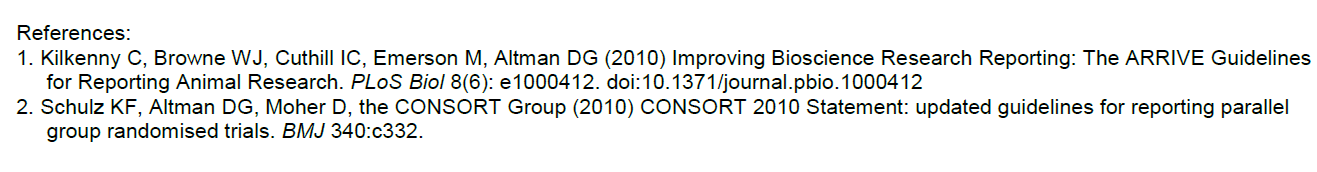

Supplement: S7 Table — (DOCX) [file pone.0258969.s007.docx]
